# Building the foundation for equitable and inclusive research: Seed grant programs to facilitate development of diverse CBPR community–academic research partnerships

**DOI:** 10.1017/cts.2022.495

**Published:** 2022-11-24

**Authors:** Chris M. Coombe, Sophia Simbeni, Aaron Neal, Alex J. Allen, Carol Gray, J. Ricardo Guzman, Richard L. Lichtenstein, Erica E. Marsh, Patricia Piechowski, Angela G. Reyes, Zachary Rowe, Julia Weinert, Barbara A. Israel

**Affiliations:** 1 School of Public Health, University of Michigan, Ann Arbor, MI, USA; 2 Department of Psychology, University of Michigan, Ann Arbor, MI, USA; 3 Chandler Park Conservancy, Detroit, MI, USA; 4 Michigan Social Health Interventions to Eliminate Disparities (MSHIELD), University of Michigan, Ann Arbor, MI, USA; 5 Community Health and Social Services, Inc., Detroit, MI, USA; 6 Michigan Institute for Clinical & Health Research (MICHR), University of Michigan, Ann Arbor, MI, USA; 7 Detroit Hispanic Development Corporation, Detroit, MI, USA; 8 Friends of Parkside, Detroit, MI, USA; 9 Poverty Solutions, University of Michigan, Ann Arbor, MI, USA

**Keywords:** Community-based participatory research, diversity, equity, and inclusion, capacity building, seed grants, community-academic partnerships, partnership development, health equity

## Abstract

**Introduction:**

The effectiveness of community-based participatory research (CBPR) partnerships to address health inequities is well documented. CBPR integrates knowledge and perspectives of diverse communities throughout the research process, following principles that emphasize trust, power sharing, co-learning, and mutual benefits. However, institutions and funders seldom provide the time and resources needed for the critical stage of equitable partnership formation and development.

**Methods:**

Since 2011, the Detroit Urban Research Center, collaborating with other entities, has promoted the development of new community–academic research partnerships through two grant programs that combine seed funding with capacity building support from community and academic instructors/mentors experienced in CBPR. Process and outcomes were evaluated using mixed methods.

**Results:**

From 2011 to 2021, 50 partnerships received grants ranging from $2,500 to $30,000, totaling $605,000. Outcomes included equitable partnership infrastructure and processes, innovative pilot research, translation of findings to interventions and policy change, dissemination to multiple audiences, new proposals and projects, and sustained community–academic research partnerships. All partnerships continued beyond the program; over half secured additional funding.

**Conclusions:**

Keys to success included participation as community–academic teams, dedicated time for partnership/relationship development, workshops to develop equity-based skills, relationships, and projects, expert community–academic instructor guidance, and connection to additional resources. Findings demonstrate that small amounts of seed funding for newly forming community–academic partnerships, paired with capacity building support, can provide essential time and resources needed to develop diverse, inclusive, equity-focused CBPR partnerships. Building such support into funding initiatives and through academic institutions can enhance impact and sustainability of translational research toward advancing health equity.

## Introduction

The effectiveness of community–academic partnerships to address health inequities is well documented, particularly when using a community-based participatory research (CBPR) approach [1,2]. Such partnerships have a commitment to equitably integrate knowledge and perspectives of diverse communities into every aspect of the research process, from identifying the research question to translating findings for change. CBPR emphasizes power sharing, co-learning, and mutual benefits for community and academic partners as they address the social determinants of health [3,4]. CBPR strives to attend to the inequities that exist in both the partnership itself and the communities involved, facilitating an anti-racist approach to research [5–8].

Despite increased calls for CBPR studies, its use remains on the margins compared to more traditional research approaches. Among the many reasons why this is the case, there are structural barriers to equity-oriented research partnerships, including systemic racial inequities in grantmaking [9]. Disparities persist in NIH research funding to African-American/Black principal investigators (PIs) and other PIs of color compared to White PIs [9,10]. Similarly, disparities exist in funding to nonprofits, with leadership diversity associated with less financial support [11]. Addressing institutional racism in grantmaking and racial power dynamics and positionalities of researchers and community partners may reduce inequities in both research and researcher–community relationships [12].

Stemming in part from structural inequities in grantmaking, of particular relevance to this article are the limited time and resources available for academic and community entities to engage in the critical stage of equitable partnership development, and lack of knowledge and skills in how to effectively conduct research in diverse partnerships [13–16]. There are few co-learning opportunities to establish partnerships prior to applying for a research grant, and most calls for proposals have a short turnaround, hindering partnership development. It is critical to enhance the capacity of and provide resources to academic and community partners to develop the knowledge and explicit processes to equitably engage diverse partners in all aspects of the research [15,17,18].

It takes time and co-learning to develop trusting and inclusive CBPR partnerships, particularly among those who have not previously worked together across race/ethnicity and other social positions, as in many predominantly White research/academic institutions working with community partners and communities of color [12,13,16]. Securing research funding for new CBPR partnerships is challenging as there is rarely compensation for the time and energy needed to establish relationships, particularly for junior faculty and community partners. Such institutional practices impede goals of diversity, equity, and inclusion (DEI) that aim to include knowledge and expertise of communities and investigators of color in research. One solution has been for funders and academic institutions to provide “seed funding” – small grants for community–academic partnerships to establish the partnership, develop methods of engaging community, and identify shared research priorities. This advances equity by decentering bias toward academics as the primary experts and sole recipients of funding.

Several studies describe models for providing seed funding alone [19,20] or in conjunction with other supports [17,21–24]. One Clinical and Translational Science Award (CTSA) provided seed grants of up to $8,000 to develop community–academic partnerships and prepare community partners to submit larger grant proposals [24]. The grants catalyzed some subsequent awards, however, many grantees had challenges sustaining partnerships, partly due to difficulty finding CBPR-trained researchers and shifting community priorities [24]. Another CTSA compared three models using small grants to build partnerships: community-initiated projects with a faculty partner; disseminating existing research to communities; and building collaborative research capacity [22]. Partnerships reported benefits of all three models; however, findings suggested there was insufficient time to sustain partnerships past initial funding. Together, these studies confirm the importance of financial and capacity building support and suggest that funding amount and duration as well partners’ preparedness can impact partnership development and sustainability.

Capacity building, such as training and technical assistance, is another mechanism to support partnership development and sustainability [18,21,24,25]. Guidance from experienced community and academic partners modeling collaborative power sharing enhances the likelihood of establishing sustainable partnerships. The Detroit Community-Academic Urban Research Center (Detroit URC) conducted a multicomponent, yearlong capacity building program for community–academic pairs that included a weeklong training, monthly forums, mentoring, and a small grant ($1,500–2,000) to develop their partnership/research project [18]. Outcomes included enhanced CBPR capacity, additional funding, sustained partnerships, and a diverse community–academic CBPR network [18]. Similarly, a CTSA provided a multicomponent CBPR training program that included pilot funds and mentorship [26]. They found that partners have varying experience doing research, securing funding, and communicating findings to the broader community, which is challenging for conducting these programs across multiple partnerships [26]. Several projects found that, while beneficial, training alone did not always secure funding, which is critical to sustaining community-engaged research partnerships [25,27].

These studies indicate that seed grants, capacity building support, or some combination of the two have the potential to foster the development of community–academic research partnerships. What is lacking is evidence that an approach that intentionally combines initial funds, capacity building, and experienced guidance from community–academic partners can contribute to the formation, effectiveness, and sustainability of equity-focused CBPR partnerships.

### Detroit Community–Academic Urban Research Center (Detroit URC) Grant Programs

To address this gap, since 2010 the Detroit URC has promoted the development of new community–academic, equity-oriented research partnerships through two small grant programs that combine seed funding with technical assistance from community and academic research partners experienced in CBPR. Established in 1995, the Detroit URC is a CBPR partnership that fosters equitable approaches to research aimed at reducing and ultimately eliminating health inequities in Detroit. The Detroit URC and affiliated partnerships, with representatives from Detroit organizations and University of Michigan (U-M) academic researchers (see Acknowledgements), aim to promote and support CBPR partnerships working to understand the relationship between the social and physical determinants of health and translate that knowledge into public health interventions and policies that build upon community resources and strengths [28].

The grant programs that are the focus of this paper have been funded and carried out by the Detroit URC in collaboration with several U-M partner entities: Poverty Solutions, Michigan Institute for Clinical and Health Research (MICHR), and U-M School of Social Work Engage Program (SSW). In this paper, we will describe and analyze the components, evaluation methods, and outcomes of these seed grant programs. We will discuss lessons learned and provide recommendations for how grant programs can facilitate the development of diverse CBPR community–academic partnerships and build the foundation for equitable and inclusionary research to advance health equity.

## Methods

### Program Design

Both the Small Planning Grant (SPG) program and the Community-Academic Research Partnerships Grant Program (C-A) were developed and overseen by the Detroit URC Board, administered by staff, and carried out by Detroit URC community–academic experts in equitable partnership research. Implementation included a call for proposals, proposal review and funding, grantee workshops, ongoing support, and formative evaluation. Grantees (community–academic teams/pairs) participated in yearlong cohorts (rounds).

### Detroit URC SPG Program

The SPG program fostered collaborative health research in Detroit by awarding partnership formation and project development seed grants to new community–academic partnerships. The program supported projects that, for example, build equitable partner relationships, explore collaborative research interests, conduct community assessments, and disseminate and translate research findings. SPG was established by the Detroit URC in 2010 through a grant from the National Institute on Minority Health and Health Disparities (NIMHD) (Grant 1RC4MD005694-01). In 2016 and 2019, respectively, MICHR and the U-M SSW partnered with the Detroit URC, increasing the number and breadth of grants offered. The SPG program provided funding up to $5000, a 2-hour grantee meeting, and technical support on request. The grantee meeting introduced teams to each other, presented a brief introduction to CBPR, and provided an overview of the program. Thirty-three projects were funded from 2011 to 2021, from $2,000 to 5,000 and averaging $4,200 per award. Over $152,000 was awarded overall.

### Community-Academic (C-A) Research Partnerships Grant Program

Modeled after the SPG, the Community-Academic Research Partnerships Grant Program (C-A) was established in 2016 as a collaboration between the Detroit URC and U-M Poverty Solutions to provide funding for collaborations between U-M academic researchers and Michigan-based community partners. Poverty Solutions is a university-wide presidential initiative aimed at partnering with communities and policymakers to find new ways to prevent and alleviate poverty through action-based research. The C-A grant program provides grants of up to $30,000 aimed to: support research focused on developing, evaluating, and strengthening programs and policies in Michigan to prevent and alleviate poverty; promote research collaboratively developed by community and academic partners; and foster community–academic partnerships and enhance their capacity to address poverty-related issues.

In addition to the larger grant amounts, C-A differed from the SPG in the type and extent of capacity building support provided, based on what was learned from SPG participants’ need for partnership development guidance. Rather than one grantee meeting, C-A grantees participated in two day-long and one half-day workshops, held in person prior to the pandemic. Facilitated by expert community and academic instructors, in addition to the content provided to SPG teams (described above), these meetings/workshops enhanced knowledge and skills, relationships, and project planning through co-learning and team working sessions. Content included partnership development and evaluation, CBPR approach to research components, and dissemination. Seventeen projects were funded from 2017 to 2021, averaging $26,600 per grant; over $453,000 was awarded. Table 1 provides an overview of both programs, including purpose, eligibility, funding, support, and grants.

**Table 1.** Characteristics of two partnership grant programs, grants, and grantees

Characteristics	Small Planning Grant Program (SPG)	Community-Academic Research Partnership Grant Program (C-A)
PROGRAM OVERVIEW:		
Collaborators and funding partners	National Institutes of Health (NIH), National Institute on Minority Health and Health Disparities, U-M Vice Provost for Global Engagement and Interdisciplinary Academic Affairs, Michigan Institute for Clinical and Health Research, U-M School of Social Work Engage Initiative, Robert Wood Johnson Foundation Clinical Scholars Program*	University of Michigan Poverty Solutions
Size of grant	$2,000–$5,000	Up to $30,000
Purpose and scope	Support new partnership formation and maintenance or project development in Detroit	Support collaborative research that aims to prevent and alleviate poverty in Michigan
Eligibility	Teams of at least one academic researcher and one Detroit-based community partner committed to engage in collaborative research	Teams of at least one U-M (any campus) academic researcher and one community partner committed to engage in collaborative research to address poverty in Michigan
Capacity building meetings/training workshops	One 2-hour grantee meeting	Two day-long grantee meetings/training workshops; third half-day meeting for Cohorts 2–4
Support	Consultation/technical assistance on request and based on review of mid-year reports	Technical assistance, trouble-shooting, additional resources on request and based on review of mid-year reports
GRANT INFORMATION:		
Dates (all grants are for duration of 1 year)	2011–2021	2017–2021
# Rounds (range of 2–5 grants per round)	11	5
# Grants	33	17
Grant funding (total $604,887)	$151,982	$452,905
Grant amount per project range	$2,000–$5,000 (plus two grants @ $10,000*)	$24,208–$30,000
Average grant amount	$4,257*	$26,641
GRANTEE PARTICIPANTS:		
# Teams (total 50)	33	17
# Participants (total 125)	71 (range 2–3 per team, average 2)	54 (range 2–5 per team, average 3)
# Academic partners (total 63)	35	28
# Community partners (total 62)	36	26
Teams with at least 1 BIPOC** partner (34 (68%) of all teams)	20 (61% of teams)	14 (82%)
# (%) Participants who are BIPOC (56 (47%) of all participants)	29 (44% of participants)	27 (50%)
Academic partners (total 22 (35%) of academic partners are BIPOC)	8 (24% of academics)	14 (50%)
Community partners (total 34 (58%) of community partners are BIPOC)	21 (64% of community)	13 (50%)
Level of experience in collaborative research (by individual participant)	Not available	*n* = 44
First experience	–	11 (25%)
Some experience	–	23 (52%)
A great deal of experience	–	10 (23%)
		
Project topics (summary)***	Healthcare (older adults, LGBTQ+, disabled, infant, homeless) Mental health Substance abuse Chronic disease management Housing Climate Green space Youth development/youth violence Research to policy translation LGBTQ outreach Older adults	Healthcare Community health Housing affordability Economic independence/economic mobility Education Food insecurity Incarceration Entrepreneurship Immigrant access to services

Source: Program documentation, pre-assessment, application. Data for this table include SPG Rounds 1–11 and C-A Rounds 1–5.

Abbreviations: U-M = University of Michigan. LBGTQ+ = lesbian, gay, transgender, and queer. BIPOC = Black, Indigenous, or People of Color.

* The Robert Wood Johnson Clinical Scholars Program funded two grants at $10,000 each in 2012 and 2013. Those two grants are included in the totals but as outliers were excluded from calculating the average grant amount.

** BIPOC (Black, Indigenous, and people of color) is an indicator of race and ethnicity, which was known for 120 of 125 participants.

*** Titles and descriptions of past grantees can be found in the Supplementary Materials and on the Detroit URC website: Detroit URC Small Planning Grants Program, and Detroit URC & U-M Poverty Solutions Community-Academic Grant Program.

### Recruitment, Application, and Selection

Using a participatory approach, the program design, request for proposals (RFP), application, and promotional materials were developed and widely disseminated with active involvement of the Detroit URC Board, workshop instructors, and previous grantees. The RFP was distributed to community and academic audiences through Board members’ and collaborators’ networks, U-M outlets, and the Detroit URC Community-Academic Research Network, composed of academic researchers and community entities interested in exploring CBPR partnerships and projects.

Applications, jointly submitted by a community–academic team, were evaluated by a diverse panel of six academic and community partners who are affiliated with the Detroit URC and have CBPR expertise and, for the C-A grants, have poverty prevention/alleviation expertise. Each application was rated independently by a community and academic reviewer, then discussed among the full panel to reach consensus, guided by CBPR principles and operating norms [28]. If needed, the panel requested additional information or suggested modifications before funding approval. A summary of proposals and review panel recommendations were submitted to the Detroit URC Board for discussion and final approval (see Supplementary Materials).

### Grantee Meetings: Capacity Building

The C-A program expanded the SPG grantee meeting by requiring all teams to participate in two full-day grantee workshops, one at the beginning and one midway through the program, and a third half-day meeting at year end, added in Round 2. Workshop objectives were for teams to share about their projects, gain understanding of CBPR, and engage in capacity building activities. Each session featured team presentations, content presentations from Detroit URC community and academic experts, team working sessions, and feedback from other grantees and community and academic instructors. At the final workshop, teams presented their findings and explored next steps for disseminating and sustaining their efforts.

At least one academic and one community partner from each team was required to attend and grantees could invite up to six additional partners. The sessions accommodated various levels of CBPR understanding and were facilitated by at least one academic and two community-based instructors, modeling equitable power-sharing relationships and processes.

### Ongoing Grantee Support

For both programs, Detroit URC staff and instructors were available on request for technical assistance, one-on-one mentoring, assistance connecting with other partnerships, and education and funding opportunities. Poverty Solutions staff provided additional support opportunities for C-A grantees. Mid-year project reports for both programs asked participants to describe any assistance needs and staff followed up accordingly.

### Evaluation Methods

Process and outcome evaluation was conducted to improve effectiveness and provide lessons learned to inform the field on using grant programs for establishing and sustaining new research partnerships. The evaluation was participatory and formative, involving Detroit URC Board members, instructors, and staff to collaboratively interpret and apply findings to support grantees and improve the program [29–31]. A concurrent integrated mixed methods design was used in which qualitative and quantitative data were collected concurrently, analyzed, fed back, and interpreted using a CBPR approach [1,32]. The study was exempt from IRB approval because it does not fit the definition of human subjects research per 45 CFR 46, 21 CFR 56.

*Program documentation and administrative data* included applications, meeting notes, participation records, and emails. A *pre-program questionnaire* was administered electronically only to C-A grantees to assess prior experience with collaborative research skills. *Progress reports* were collected for both grant programs. At 6 months, each grantee team submitted a narrative *mid-way progress report* to discuss objectives, collaborative approach, challenges, future plans, budget, and assistance needed. A narrative *final report*, submitted 1 month after the program year ended, asked about: outcomes, accomplishments, and impacts; lessons learned; publication/dissemination plans; funding; and intention/actions to sustain the project and/or partnership beyond the grant. All grantee meetings were evaluated by attendance, observation, and an *assessment questionnaire* administered at the end of each meeting. Closed-ended questions assessed meeting effectiveness and usefulness. Open-ended questions asked about what was most and least valuable, recommendations, and additional learning needs/interests.

To further assess outcomes and accomplishments, a *follow-up/post-questionnaire* was administered through Qualtrics survey software to multiple rounds of grantees between 1 and 3 years after project completion. Questions included the status of the partnership and project (e.g., continuing and completed), outcomes/accomplishments (e.g., relationships built, research conducted, proposals submitted, funding received, and dissemination), and impact on the community. The questionnaire was administered to SPG in 2014 (Rounds 1–3), 2018 (Rounds 5–8), and 2021 (Rounds 9–10). SPG Round 4 projects were not surveyed due to staffing limitations. The questionnaire was administered to C-A in 2021 (Rounds 1–3). When the 2021 survey was administered, five SPG teams (Round 11) and five C-A teams (Rounds 4–5) had not completed their projects largely due to pandemic-related delays and were not included.

### Data analysis, feedback, interpretation, and application

Descriptive statistics were compiled and analyzed for closed-ended questions and to quantify selected qualitative data (e.g., grants and publications). Open-ended responses were organized into smaller, meaningful data pieces, and common themes were identified remaining close to participants’ own words to preserve meaning [33]. Quotes were identified to illustrate findings. The follow-up outcomes/accomplishments question differed somewhat between administrations, with the 2014 and 2018 questionnaires asking “To what extent did the partnership accomplish the following” rated on a five-point Likert scale from “None” to “To a Great Extent.” The 2021 questionnaire asked “Which of the following did your partnership accomplish? Check all that apply.” For comparability in this paper, we dichotomized the earlier questionnaire responses, coding “Somewhat” and “To a great extent” as Yes.

All findings were compiled and presented to instructors and staff to inform program implementation. Staff and instructors reviewed progress reports to identify and respond to challenges or requests for assistance (see Supplementary Material for evaluation instruments).

## Results

### Characteristics of Grants and Grantees

Table 1 provides summary information for both grant programs. From 2011 to 2021, fifty community–academic teams received collaborative research grants. Two-thirds (33) were SPG partnership/project development grants, averaging $4,257, and one-third (17) were C-A research grants, averaging $26,641. Of the total $604,887 awarded, one-fourth ($151,982) were for the smaller SPG grants and three-fourths ($452,905) for the larger C-A poverty-specific grants.

All teams were composed of at least one academic and one community partner totaling 125 participants, approximately half of each, and averaged two to three members per team (SPG and C-A, respectively). Of the C-A participants who completed the pre-assessment, 77% had no or some previous experience in collaborative research. For SPG grantees, data on previous experience were not available; however, only new/newly developing partnerships were eligible.

Participants were racially and ethnically diverse, with nearly half identified as Black, Indigenous, or People of Color (BIPOC): 44% of SPG and 50% of C-A participants. Overall, 61% (20) of SPG teams and 82% (14) of C-A teams had at least one BIPOC partner, and 36% of academic and 58% of community partners were BIPOC individuals. For SPG teams about a fourth of academics and two-thirds of community partners were BIPOC, while for the C-A teams half of academic and half of community partners were BIPOC individuals.

Participants in both programs were from multiple disciplines (e.g., public health, urban planning, and law). Team projects addressed diverse determinants of health (e.g., housing affordability, technology, economic self-sufficiency, and climate resilience). Approaches addressed multiple levels of change including individual, programs/interventions, organizational, systems, and policies (see Supplementary Materials for grant recipients).

### C-A Grantee Meetings Evaluation

Table 2 shows quantitative results from all grantee meeting evaluations. Twelve grantee meetings were held across five C-A rounds, with two meetings each for Rounds 1 and 5 and three meetings for rounds 2–4. Overall, 115 evaluation questionnaires were completed and compiled across all meetings and included each participant evaluating more than one meeting. Presentation topics and working sessions differed in content between each of the two to three meetings per round, and topic-specific items were only included in that session’s questionnaire. Hence, there were fewer responses for session-specific items, particularly for the third meeting which was held for only three of the five rounds. Participants rated aspects of the grantee meetings by indicating agreement on a five-point scale, for example, from strongly disagree to strongly agree.

**Table 2.** Grantee Meetings evaluation results for the Community-Academic Research Partnership Grant Program (all meetings for Rounds 1–5 combined)*

Please indicate your level of agreement with the following statements about the Grantee Meeting:	Strongly Disagree/Disagree	Neither Agree nor Disagree	Agree/Strongly Agree
Overall the meeting content and structure was well organized (*n* = 115).	1 (1%)	1 (1%)	113 (98%)
Meeting facilitators demonstrated expertise in the subject matter (*n* = 115).	1 (1%)	4 (3%)	110 (96%)
Learning resources (binder, handouts, and resource list) will be useful to our partnership in the future (*n* = 114).	3 (3%)	7 (6%)	104 (91%)
Interactive exercises and questions were used effectively (*n* = 114).	3 (3%)	4 (3%)	107 (94%)
Opportunities for our team to work together on specific tasks were valuable (*n* = 111).	1 (1%)	3 (3%)	107 (96%)
Learning about and networking with other partnerships was beneficial to our work (*n* = 114).	3 (3%)	14 (12%)	97 (85%)
The presentation on the following topic was useful:			
Meeting 1: CBPR and partnership development (*n* = 48)	0 (0%)	1 (2%)	47 (98%)
Meeting 1: Partnership evaluation (*n* = 48)	0 (0%)	5 (10%)	43 (90%)
Meeting 2: Collaborative data interpretation (*n* = 49)	1 (2%)	4 (8%)	44 (90%)
Meeting 2: Dissemination of partnership findings (*n* = 49)	0 (0%)	1 (2%)	48 (98%)
Meeting 3: Developing a persuasive message (*n* = 11)	0 (0%)	4 (36%)	7 (64%)
Please indicate how useful the team working sessions were for your partnership in each of the following areas:	Not at all Useful/Somewhat Useful	Moderately Useful	Very Useful/Extremely Useful
Partnership development – Meeting 1 (*n* = 44)	3 (7%)	5 (11%)	36 (82%)
Planning next steps for your partnership – Meeting 2 (*n* = 40)	0 (0%)	2 (5%)	38 (95%)
Developing working relationships among your team – Meeting 1 (*n* = 43)	2 (5%)	2 (5%)	39 (90%)
Developing working relationships among your team – Meeting 2 (*n* = 50)	1 (2%)	5 (10%)	44 (88%)
Developing working relationships among your team – Meeting 3 (*n* = 11)	0 (0%)	1 (9%)	10 (91%)
Reflection on team presentations and feedback – Meeting 2 (*n* = 51)	0 (0%)	7 (14%)	44 (86%)
Reflection on team presentations and feedback – Meeting 3 (*n* = 15)	0 (0%)	2 (13%)	13 (87%)
Data collection planning – Meeting 1 (*n* = 38)	2 (5%)	6 (16%)	30 (79%)
Evaluating your partnership – Meeting 1 (*n* = 45)	3 (6%)	7 (16%)	35 (78%)
Challenges and facilitating factors – Meeting 1 (*n* = 35)	1 (3%)	3 (8%)	31 (89%)
Data feedback and interpretation – Meeting 2 (*n* = 51)	0 (0%)	7 (14%)	44 (86%)
Dissemination of findings, developing dissemination guidelines – Meeting 2 (*n* = 51)	1 (2%)	8 (16%)	42 (82%)
Developing a persuasive message: The 27-9-3 activity – Meeting 3 (*n* = 8)	0 (0%)	0 (0%)	8 (100%)

* Total *N* = 115 represents completed questionnaires from all 12 meetings across 5 rounds. Thus, *n* includes responses from the same participants evaluating different meetings. Because content differed between meetings 1 and 3, questions that were specific to each meeting have the n listed following the item. Smaller *n* values for individual evaluation items are noted in the table and are indicative of partial participation in evaluation surveys throughout rounds or between meetings.

CBPR = Community-based participatory research.

The C-A grantee meetings were rated highly (>90% agreement) on content, organization, instructor expertise, learning resources, and effectiveness of activities. Over 90% found the CBPR presentations useful. Team working sessions with instructor consultation were rated very/extremely useful by most participants and contributed to the development of working relationships among team members (88–91%). Planning their partnership’s next steps was rated most highly (95%). Learning about and networking with other teams was considered beneficial by 85% of participants. Sessions with somewhat lower usefulness ratings (78–86%) addressed content that partnerships were not yet engaged in (e.g., data collection and feedback, dissemination, and partnership evaluation). The session on persuasive messages was conducted at the end of the third meeting with few participants remaining, and four of the seven survey respondents neither agreed nor disagreed regarding usefulness.

Participants’ positive assessment was further explained from open-ended questions, presented in Table 3 as summary themes and illustrative quotes. C-A participants’ descriptions of what was most valuable/beneficial fell into seven major themes.

**Table 3.** Most valuable/beneficial aspects of Community-Academic Grant Program grantee meetings: themes and illustrative quotes from evaluation surveys

Major themes	Illustrative quotes
Theme 1. Having dedicated time to come together as a team to reflect on our work and plan next steps	The opportunity to meet in person with our partners and focus on specific aspects of the project. It is valuable to be able to reflect as a team. We do many things in the project, so it is nice to be able to stop and reflect. A chance to deeply reflect on our work and make plans for moving forward. Having an opportunity to come together as a team to work through some of our most pressing questions and next steps.
Theme 2. Time to focus on partnership development (relationships, group process, and evaluation)	Just having time and space to work *on* the partnership instead of *in* the partnership. Time to focus on the partnership and reflect on what is working and where we need to make adjustments. Having the time to talk about planning the process whereas usually we heavily cover content. Putting our work in a larger context. Step back and recognize/acknowledge that we are in a working partnership with a process that we can evaluate and improve.
Theme 3. Structured team working/planning sessions with guided activities and facilitator feedback	Guided group discussion questions with supportive coach/facilitator. Time spent with the team to think through questions. The individual team discussion sessions were useful for clarifying next steps for our partnerships and how to involve community partners in interpretation. Simply having the meeting as a check-in and way to plan partnerships going forward (e.g., thinking through dissemination plans).
Theme 4. Learning about CBPR from highly experienced community and academic partners as presenters and instructors	The presenters are all so knowledgeable and present the info in engaging ways; the real-life examples and their personal experiences are helpful. A facilitator sitting w/our group during our work sessions to offer support and play devil’s advocate. The expert panel was terrific. Community presenters were awesome! Enlightening to hear about other projects, experiences, and lessons learned from facilitators. All the facilitators were remarkably warm and genuinely interested in us.
Theme 5. Gaining knowledge and understanding of CBPR processes and approach to carrying out research	Understanding how CBPR can successfully work. We were essentially walked through – as a group how we can implement a more focused approach towards our CBPR work. Being reminded of the importance of community engagement throughout the life of the project. I have not personally been a part of a research study; sharing information about the basics of how to analyze data and disseminate it (“for dummies”) was helpful. [The community instructor] stretched our thinking on how to activate findings by suggesting we consider how our results might influence policy.
Theme 6. Exchanging ideas and learning from experiences of other teams	I didn't know we had so much to say until asked to present! The opportunity to hear progress from other teams and receive their excellent suggestions on the further development of our project. Hearing suggestions from other grantees, getting an “outsider’s” perspective. The potential for cross-trainings and working with other groups where our research intersects. I appreciated connecting with the other awardees. I like the idea of being connected to a group of scholars and practitioners working on important topics.
Theme 7. Well-balanced organization, structure, process, and content	It’s wonderful to have a structure and process for thinking through this work. The grantees meetings have been well organized, structured, and delivered. They are enjoyable, informative, and enriching to my research. Resources/info/presentation in conjunction with team planning and action planning were paired and well planned!! I think it’s a great balance of tasks, discussion, + slides. I mean really A + balance. I think it’s very useful to see the support and infrastructure for the Detroit URC that is at our disposal as grantees on this project. This has been a great experience that moved our project and our partnership forward!

### Theme 1. Dedicated time to come together as a team

Cutting across all themes, participants highly valued setting aside a substantial block of time to meet as a team.

### Theme 2. Focus on partnership development

Teams highly valued the specific focus on partnership development, including how they will work together, what’s working and what needs adjustment, and stepping back to remember that the relationship is central.

### Theme 3. Structured team working/planning sessions

Having dedicated time and processes for working together provided opportunities to apply CBPR to their research project. Guided activities enabled them to collaboratively work through pressing issues and plan for next steps.

### Theme 4. Highly experienced community and academic presenters/instructors

Experienced community and academic instructors in the working sessions were supportive coaches, providing models for using CBPR, posing questions, and suggesting methods others have used.

### Theme 5. Understanding and knowledge of CBPR processes and approach

Being walked through the research process using CBPR was beneficial to both academics, for example, how to involve communities throughout, and community partners, to familiarize themselves with research methods. Presenters provided real-life examples and a safe space for questions.

### Theme 6. Exchanging ideas and learning from other teams

Participants valued hearing about other teams’ projects and progress, and getting “outsider” feedback, ideas, and perspectives. Connecting with other grantees opened potential areas of intersection for their work.

### Theme 7. Overall organization, structure, process, and content

Participants described the meetings as well organized and delivered, with a beneficial balance of presentation, discussion, team planning, and action-oriented activities. Meetings were enjoyable and moved their partnerships forward.

Participants described several areas as least valuable and recommended changes that instructors considered for subsequent sessions. These included wanting more interactive presentations/fewer slides, changing the length of workshops (some requested more time, others less), and more time working in teams to plan and apply content.

Overall, participants expressed satisfaction with the integration of process and content and comments were quite positive, as one participant stated: “The grantees meetings…are enjoyable, informative, and enriching to my research.” Another summarized, “This has been a great experience that moved our project and our partnership forward!”

### Ongoing Grantee Support

Support provided on request or proactively for both programs included guidance on partnership development, funding, partner identification, and dissemination. Specific examples included assistance hiring a research assistant, widespread publicity of research findings to inform public opinion on a proposed rule, and professional video production. Both the Detroit URC and Poverty Solutions publicized accomplishments through their newsletters, websites, news venues, and conferences.

### Outcomes, Accomplishments, and Impact of Both Grant Programs

Outcomes and accomplishments were assessed along several dimensions, including: CBPR partnership development and capacity; research findings and dissemination; interventions, systems, and policy translation; and funding and sustainability. Table 4 summarizes quantitative results from the follow-up/post-questionnaire administered to grantees 1–3 years after completion. Additional data from open-ended survey questions and progress reports (e.g., grants, dissemination activities, and quotes) are reported below but not in Table 4.

**Table 4.** Partnership outcomes and accomplishments as a result of the grant programs reported 1–3 years following completion of the program (N = 32 of 36 grantee teams surveyed*)

Partnership accomplished the following as a result of the grant program**	SPG 2011–2018 Rounds 1–3, 5–8 (17 of 17 teams) *n* (%)**	SPG 2019–2020 Rounds 9–10 (5 of 7 teams) *n* (%)	C-A 2017–2020 Rounds 1–3 (10 of 12 teams) *n* (%)
Built relationships between partners	17 (100)	4 (80)	10 (100)
Developed a steering committee or partnership infrastructure mechanism	13 (76)	4 (80)	6 (60)
Evaluated partnership process	10 (59)	1 (20)	0
Conducted new research	14 (80)	3 (60)	8 (80)
Analyzed data	15 (88)	3 (60)	8 (80)
Submitted proposals for funding	15 (88)	2 (40)	3 (30)
Received additional funding***	9 (53)	1 (20)	10 (100)
Disseminated or translated research findings***	12 (71)	2 (40)	10 (100)
Planned/explored future research collaboration	16 (94)	1 (20)	6 (60)
Continuation of partnership	17 (100)	5 (100)	10 (100)

SPG = Small Planning Grant Program. C-A = Community Academic Research Partnership Grant Program.

* 32 of 36 grantee teams who submitted final project reports between 2017 and 2020 completed the follow-up/post-questionnaire.

** Number and percent of teams that responded affirmatively (see Methods for question wording and response categories).

*** Questionnaire data on funding and dissemination were supplemented with information provided in progress reports.

Of teams surveyed, 89% (32 of 36) completed the questionnaire: 92% (22 of 24) of SPG teams and 83% (10 of 12) of C-A teams. Data are combined for the SPG 2014 and 2018 surveys and reported separately for the 2021 survey of SPG Rounds 9–10 (2019–2020) and C-A Rounds 1–3 (2017–2020), which included pandemic-related program delays.

*Partnership development and capacity building*. Twenty of 22 SPG grantees and all 10 C-A grantees reported that they built or strengthened relationships during the program. Over three-fourths of SPG and 60% of C-A teams developed a formal partnership structure. Fewer grantees reported evaluating the partnership; about half of SPG teams and no C-A teams. All teams reported continuing the partnership on the same or a different project. Among the earlier SPG rounds, 92% explored future research collaborations. Among those surveyed in 2021, 20% (one of five) SPG grantees and 60% (six of ten) C-A grantees explored future collaborations. Grantees cited partnership development as contributing to sustainability: “What this grant really facilitated was the development of the partnership, specifically the relationship building. It helped us set the groundwork where we were then able to do a first research study that was funded through other sources.” (Also see *Sustainability*, below.)

*Innovative research, data, and dissemination.* Although some SPG teams received partnership development rather than project development grants, over 81% of all teams conducted research, pilot projects, and/or analyzed data during the grant period, comparable between SPG (82%) and C-A (80%) grantees. Two-thirds of SPG grantees and all C-A grantees disseminated and/or translated research findings. Two C-A teams produced publicly accessible data sets to inform administrative and policy decision-making on housing affordability. Innovative methods included use of text messaging to evaluate perceptions of health services (SPG grantee), and development of an animated video to train staff and increase participation in a foreclosure prevention program (C-A grantee). Results were disseminated through peer reviewed publications, policy briefs, conference presentations, community forums, news articles, and social media.

*Interventions, systems, and policy impacts.* Both programs funded projects with translational objectives, conducting research to evaluate and strengthen interventions, programs, and/or policies. Several teams were able to quickly mobilize their partnership to address urgent public health needs:

“After the onset of the COVID-19 pandemic, our data and expertise were used to collaborate with the grant program in advocating for a statewide eviction moratorium. We formalized and conducted a follow-up policy report with the same team, providing a rapid synthesis and policy recommendations about pandemic-related eviction policies.”

Table 5 presents selected examples from progress reports of accomplishments, outcomes, and impacts at multiple levels: partnership development and capacity building; innovative research, data, or dissemination; intervention or program development/evaluation; and policies or systems change. Examples represent multiple communities, health determinants, issues, and strategies.

**Table 5.** Selected examples* of accomplishments, outcomes, and impacts of grant projects at multiple levels

Grant project	Accomplishments, outcomes, and impact (from mid-year and final progress reports)
1. Partnership Development and Capacity Building	
Preventing Tax Foreclosure (C-A Round 1)	Team developed training tools and a video explaining property tax foreclosure prevention to increase staff capacity to provide assistance, lessen staff burden, and improve access.
Neighborhood-Based Community Health Worker Initiative to Improve Health and Strengthen Community Capacity (C-A Round 1)	Community health workers (CHWs) in a health system within the local community were trained to build capacity for new CHWs.
Detroit Urban Native Health Collaborative (SPG Round 3)	The Detroit Urban Native Health Collaborative hosted a half-day CBPR training to discuss how Native American communities may equitably engage in research with university partners.
Detroit Climate Action Collaborative Climate Change Action Plan (SPG Round 2)	Team strategically identified partners for a steering committee to guide development of an evidence-based Climate Action Plan and prepared to launch a community engagement program.
2. Innovative Research, Data, and Dissemination	
Preventing Tax Foreclosure (C-A Round 1) Preserving Low-Income Housing (C-A Round 1)	Two separate projects developed datasets that include difficult-to-access data related to housing affordability policies to analyze and monitor policy implementation to protect access to affordable housing (property tax exemption and low income housing tax credits).
Youth Experiences of Violence, Discrimination and Harassment (SPG Round 1)	Team assembled a Youth Advisory Board that developed and conducted a photo voice project on causes and prevention of violence across three communities in Detroit. Held a community forum to present results to local elected officials, with photo exhibit, workshop, performances, film, and discussion.
Chilling Impact of the Public Charge Rule on Immigrants’ Access to Services (C-A Round 2)	Team found that proposed changes to the public charge rule contributed to an anti-immigrant climate and made immigrants fearful of accessing services, being out in public. Results were widely disseminated through op-ed and articles to engage the public.
3. Intervention or Program (development and evaluation)	
Neighborhood-Based Community Health Worker Initiative to Improve Health and Strengthen Community Capacity (C-A Round 1)	A health plan, community partners, and community health workers jointly developed a community health worker intervention that met needs of health plans and the health needs of the community.
Detroit Urban Native Health Collaborative (SPG Round 3)	Team was awarded an implementation grant from Substance Abuse and Mental Health Services Administration which created opportunities to expand and implement the community organization’s culturally responsive services.
Opportunity Not Punishment: Pilot Functional Sentencing Program (C-A Round 3)	Successfully assisted a nonprofit organization in Detroit, Michigan in implementing and evaluating a pilot functional sentencing program in the 36th District Court in Detroit.
Recipe for Success Cooking Program (C-A Round 3)	Piloted and evaluated a new cooking class program in a community health center to increase participation in existing center programs and build core skills to decrease food insecurity among low-income patients.
4. Policy or Systems Change	
Opportunity Not Punishment: Pilot Functional Sentencing Program (C-A Round 3)	Three judges implemented a functional sentencing (FS) pilot, to help individuals permanently exit the criminal justice system by replacing fines and costs with targeted interventions (e.g., job placement and medical services) that address the root causes of an individual’s offense. Met with court staff to implement FS principles into probation services, and with judicial authorities in other jurisdictions in SE Michigan who are considering adopting the program.
Michigan Evictions: Assessing Data Sources and Exploring Determinants (C-A Round 3)	Researched the rate and impact of evictions in Michigan and shared findings with key legal and planning organizations, court administrators, and judges who influence eviction policies and decisions. After the onset of the COVID-19 pandemic, data and expertise were used to advocate for a statewide eviction moratorium, and a policy report was produced to provide a rapid synthesis and policy recommendations about pandemic-related eviction policies.
Preventing Tax Foreclosure (C-A Round 1) Preserving Low-Income Housing (C-A Round 1)	In two separate projects, previously unavailable housing affordability data (property tax exemption and low-income housing tax credits) were compiled and translated into reports/policy documents to inform procedures and policies (e.g., lawsuit settlement terms, city ordinance, changes to tax exemption application, and strategic plan to preserve affordable housing).
Collaborative Approach to Community-based Research on Breastfeeding (SPG Round 6)	Project worked with the National Black Breastfeeding Caucus and other national advocacy organizations on a comprehensive Call to Action to align community activism, interdisciplinary academic support, and policy development to increase effective breastfeeding support for black mothers. Partners explored collaborations with child daycares and others to advance policy driven, social, and other support for breastfeeding among African American women.

* Data are from grantee mid-year and final project reports. Grant project examples were selected to represent a range of communities, health determinants, issues, and strategies.

C-A = Community Academic Research Partnership Grant Program. SPG = Small Planning Grant Program.

*Sustainability, additional funding, and expanded collaborations*. In addition to partnerships continuing, 88% of SPG grantees submitted funding proposals and nearly half received funding ranging from $1,200 to $300,000. All C-A grantees received additional funding. Funders included Poverty Solutions, academic institutions/entities, foundations, corporations, and federal institutes. Several projects received large-scale funding and attributed it to the grant program.

“Earlier this year…we were funded by NIH for an R01, in partnership with [our partner], to test an intervention facilitated by community health workers. We believe that the URC-funded work played a significant role in our success! We have been working together closely these past few months to launch the study. Thank you for your critical support.” – SPG Academic partner

## Discussion

Engaging communities in equity-focused and translational research and promoting development of community–academic partnerships using a CBPR approach can advance health equity. Providing the time, resources, and capacity building support for newly forming partnerships to build relationships, infrastructure, and power sharing processes is crucial to establishing inclusive, equitable research partnerships. This study described two grant programs that promote the formation of such partnerships through seed funding combined with capacity building support from expert community and academic instructors.

Over 10 years, the programs fostered the development of 50 collaborative partnerships that were diverse along multiple dimensions. Nearly half of participants were persons of color (BIPOC), and two-thirds of teams had at least one BIPOC member. While among earlier grantees academics were disproportionally White, the C-A teams had equal numbers of community and academic partners who were BIPOC.

Participating as community–academic teams laid a foundation of joint ownership, co-learning, and power sharing. Throughout the year, both academic and community partners built their capacity for engaging communities in all aspects of the research. Academic partners established or deepened their connections with the community both in and outside of the project, furthering sustainability. Grantee meetings provided a rare opportunity for teams to have extended planning time together, with guidance from community and academic experts. Exchanging ideas in person provided a powerful way for teams to build research relationships and capacity to speak their minds across differences while developing their infrastructure and research plans. As one participant noted, “Having an opportunity to come together as a team to work through some of our most pressing questions and next steps was most valuable…It’s wonderful to have a structure and process for thinking through this work.”

All partnerships continued collaborating beyond the year, which is a substantial accomplishment among seed grant programs. At the time of this writing, many participants (individuals and teams) continued to engage in collaborative research or using the findings for action, demonstrating the sustainability of the equitable research capacity and relationships built through these programs. A central aim of both CBPR and translational science is to apply findings to achieve beneficial impact/bring about change [34,35]. Outcomes included: innovative research; application of findings to intervention, systems, or policy change; dissemination; and additional funding and collaborations [36,37].

The diverse, multi- and transdisciplinary approach fostered knowledge and collaboration among grantees, and the broader networks and initiatives of the program collaborators/funders provided linkages to other opportunities. Most teams successfully leveraged this initial grant to secure additional resources to enhance and/or sustain their partnerships.

These findings are consistent with those of other seed grant approaches to engaging communities in research and reinforce the importance of funding, capacity building support, and time to develop diverse collaborative partnerships. However, our findings differ from studies which found modest outcomes from small compared to somewhat larger grants, and difficulty sustaining partnerships [22]. Our findings demonstrated that research resulting from even small amounts of funding had an impact on establishing trusting relationships, applying findings to benefit communities, and sustaining partnerships for future endeavors. Although the two grant programs described here differed in several ways, most notably in the amount of funding and capacity building support, both were similarly effective in building ongoing relationships and continued engagement in collaborative research by at least one partner. However, the C-A larger funding amount and explicit focus on a research outcome may have contributed to more substantive research findings and impacts.

### Recommendations

We recommend the following to those seeking to use seed funding strategies to build or enhance community–academic research partnerships for advancing equity.

1. Partnership development is a necessary initial phase of conducting equitable, inclusionary research. Dedicated funding, time, and capacity building support for partnership development is essential for forming and sustaining community–academic research efforts. Most grantees requested a 6-month no-cost extension, suggesting that similar grant programs consider at least 18 months rather than 1 year duration.
2. To promote equity, grant programs should integrate a co-learning and power sharing approach with mutual ownership and benefits. Supporting community–academic teams rather than individuals and ensuring that grantees are racially and ethnically diverse can counteract existing structural inequities.
3. Substantive, structured capacity building activities promote inclusivity and enable individuals and the partnership to develop equity-based skills, processes, and relationships while planning their research. Racially/ethnically diverse community and academic instructors discussing the research process from their perspectives provides an open learning environment and demonstrates trusting relationships.
4. Institutional linkages to resources, opportunities, and networks can enhance impact, sustainability, and greater equity in research support, particularly for BIPOC academics and communities. Universities aspiring to promote DEI and counteract internalized and racist research practices must incentivize researchers to conduct more equitable research and dedicate resources to practices that address power differences. Institutions should provide opportunities for new partnerships to secure funding, disseminate findings, sustain the partnership, and expand collaborations – with explicit assurance of equitable and dedicated funding for the communities involved.
5. Finally, we strongly encourage NIH and foundations to build substantive funds and time for partnership development into their funding initiatives, thus enhancing effectiveness, sustainability, and impact. Initial SPG funding was from NIMHD, demonstrating that relatively small, time-limited funding and capacity building for community–academic research can have important impacts. However, short-term projects need longer-term support to effectively translate findings into interventions, systems, and policies to strengthen impact. We recommend that NIH implement a multiphase funding process to support CBPR partnerships similar to one by NIMHD, which funded CBPR partnerships in three phases: partnership development (3 years); intervention implementation (5 years); and dissemination (3 years).

### Participants Affirmed these Recommendations

“Formalizing our partnership and having a funding pool to enable our project made all the difference. We would not have been able to do this without it, and the project has led to so many other productive activities around the issue and others.”

*Limitations*: Initially funded for 2 years, the SPG was sustained through a patchwork of funding with limited evaluation resources. Grantees from both programs were surveyed 1 to 3 years following completion, and most grantees requested no-cost extensions for project continuation, extending time without funds. Because the outcomes/accomplishments response categories differed between administrations, earlier scaled data were dichotomized resulting in potential loss of information, which, however, would not diminish results. After 2019, the pandemic substantially interrupted work on projects and grantee meetings which may have affected outcomes.

## Conclusion

These findings demonstrate that a small amount of seed funding paired with technical assistance and capacity building for newly forming community–university partnerships can provide the time and resources needed to establish the trust required for developing and sustaining successful CBPR partnerships. We hope that this analysis will provide guidance for other seed grant programs, and to federal funders and foundations committed to fostering CBPR partnerships toward diversity, inclusion, and equity.

## Supplementary material

For supplementary material accompanying this paper visit https://doi.org/10.1017/cts.2022.495. click here to view supplementary material
