# Building the foundation for equitable and inclusive research: Seed grant programs to facilitate development of diverse CBPR community-academic research partnerships – CORRIGENDUM

**DOI:** 10.1017/cts.2023.636

**Published:** 2023-10-25

**Authors:** Chris M. Coombe, Sophia Simbeni, Aaron Neal, Alex J. Allen, Carol Gray, J. Ricardo Guzman, Richard L. Lichtenstein, Erica E. Marsh, Patricia Piechowski, Angela G. Reyes, Zachary Rowe, Julia Weinert, Barbara A. Israel

In the original version of this article, reference 13 in the references list had the incorrect DOI listed against it.

This has now been updated to doi: 10.1177/14687941211065163.

The original article has been corrected online to rectify this error.
