# Supplementary material for: Building the foundation for equitable and inclusive research: Seed grant programs to facilitate development of diverse CBPR community–academic research partnerships
Source: J Clin Transl Sci. 2022 Nov 24;7(1):e2. doi: 10.1017/cts.2022.495 (PMC9879886; doi:10.1017/cts.2022.495)
Supplement: Supplementary file 1 [file S2059866122004952sup001.zip › S2059866122004952sup001.pdf]

Community-Academic Partnership Seed Grant Programs* to Build the Foundation for Equitable and Inclusive Research

Supplementary Materials – Table of Contents

1. Program Timeline - C-A
2. Application, Review, and Selection
    2.1. Request for Proposals
        2.1.1. Request for Proposals - SPG
        2.1.2. Request for Proposals - C-A
    2.2. Review Process Description - C-A
    2.3. Reviewer Scoring Sheet - SPG
3. List of Grant Recipients
    3.1. Grant Recipients - SPG
    3.2. Grant Recipients - C-A
4. Evaluation Instruments
    4.1. Pre-Assessment - C-A
    4.2. Grantee Meeting Assessment Questionnaire - C-A
    4.3. Mid-Way Progress Report Guidelines
        4.3.1. 6 Month Progress Report - SPG
        4.3.2. 6 Month Progress Report - C-A
    4.4. Final Report Guidelines - C-A
    4.5. Follow-Up Questionnaire - C-A

*SPG: Small Planning Grant Program
C-A: Community Academic Research Partnerships Grant Program
