# Supplementary material for: Building the foundation for equitable and inclusive research: Seed grant programs to facilitate development of diverse CBPR community–academic research partnerships
Source: J Clin Transl Sci. 2022 Nov 24;7(1):e2. doi: 10.1017/cts.2022.495 (PMC9879886; doi:10.1017/cts.2022.495)
Supplement: Supplementary file 1 [file S2059866122004952sup001.zip › S2059866122004952sup002.pdf]

Detroit URC Small Planning Grant Review Form

Directions: The following review criteria will be used to score the Detroit URC Small Planning Grant Proposals.

Rate each criterion on a scale from 1-5:

1. Very Weak (lowest score)
2. Weak
3. Adequate
4. Strong
5. Very Strong (best score)

If there is not sufficient evidence to score the proposal on a particular criterion, please check "Not Able to Assess." Please use the ‘notes’ space below each criterion to explain and justify the score you gave. You may also use the space to write questions or suggestions. (Notes are optional.)

Please review all the applicant’s submitted materials prior to filling out the scoring sheet.

Project Leads:

Name of Reviewer:

Q1. Evidence of the rationale for the need for this collaborative partnership

1. Very Weak	2. Weak	3. Adequate	4. Strong	5. Very Strong	Not Able to Assess

Comments:

Q2. Evidence of responsiveness to the community partner's needs/interests

1. Very Weak	2. Weak	3. Adequate	4. Strong	5. Very Strong	Not Able to Assess

Comments:

Q3. Extent and feasibility of academic partner involvement

1. Very Weak	2. Weak	3. Adequate	4. Strong	5. Very Strong	Not Able to Assess

Comments:

Q4. Extent and feasibility of community partner involvement

1. Very Weak	2. Weak	3. Adequate	4. Strong	5. Very Strong	Not Able to Assess

Comments:

Q5. Potential for future research collaboration

1. Very Weak	2. Weak	3. Adequate	4. Strong	5. Very Strong	Not Able to Assess

Comments:

Q6. Potential of funding for future collaborative research efforts

1. Very Weak	2. Weak	3. Adequate	4. Strong	5. Very Strong	Not Able to Assess

Comments:

Q7. The timeline is clear, detailed, and reasonable.

1. Very Weak	2. Weak	3. Adequate	4. Strong	5. Very Strong	Not Able to Assess

Comments:

Q8. The budget is clear, detailed, and realistic.

1. Very Weak	2. Weak	3. Adequate	4. Strong	5. Very Strong	Not Able to Assess

Comments:

Q9. Evidence that the budget has been developed and discussed by all partners and allocated equitably as appropriate.

1. Very Weak	2. Weak	3. Adequate	4. Strong	5. Very Strong	Not Able to Assess

Comments:

Q10. Letters of commitment are sufficient.

1. Very Weak	2. Weak	3. Adequate	4. Strong	5. Very Strong	Not Able to Assess

Comments:

Q11. Overall, how would you score this proposal?

1. Very Weak	2. Weak	3. Adequate	4. Strong	5. Very Strong	Not Able to Assess

Comments:

Q12: Do you recommend this proposal for funding?

Highly Recommend	Recommend	Do Not Recommend

Direct questions to:
Detroit URC Center Manager
detroiturc@umich.edu
