# Supplementary material for: Building the foundation for equitable and inclusive research: Seed grant programs to facilitate development of diverse CBPR community–academic research partnerships
Source: J Clin Transl Sci. 2022 Nov 24;7(1):e2. doi: 10.1017/cts.2022.495 (PMC9879886; doi:10.1017/cts.2022.495)
Supplement: Supplementary file 1 [file S2059866122004952sup001.zip › S2059866122004952sup003.pdf]

Community-Academic Research Partnerships

2019-2021 Timeline of Activities

Program Development/Revisions (August - September, 2019)

- ☐ Revise program guidelines
- ☐ Revise application process (e.g., call for proposals, application, application criteria, deadlines, review process)
- ☐ Develop and revise promotional materials
- ☐ Invite participants to sit on the application review committee
- ☐ Determine collaborative research capacity building training structure/components
- ☐ Announce and publicize the program

Program Launch (October - November, 2019)

- ☐ Continue to publicize the program (e.g., websites, Community-Academic Research Network eNewsletter, U-M faculty group listservs, community organization networks)
- ☐ Respond to questions from and consult with applicants
- ☐ Refine application review process (revise/finalize review criteria and scoring process)
- ☐ Accept applications by stated deadline and close request for proposals
- ☐ Further develop/revise collaborative research capacity building curriculum and evaluation plan
- ☐ Identify trainers for capacity building workshop

Grant Review Process (November - December, 2019)

- ☐ Accept and triage applications
- ☐ Distribute applications to review committee for review
- ☐ Compile review committee scores
- ☐ Convene review committee to make funding decisions
- ☐ Notify applicants of award decisions in early December
- ☐ Announce and publicize grant awards (mid-late January)

1st Half of Grant Program Period (January - July, 2019)

- ☐ First collaborative research day-long training conducted and evaluated
- ☐ Funds administered and distributed (Award start date January 1, 2020)
- ☐ Grantees begin implementation of collaborative research project
- ☐ Publicity/Profiles of each grantee team developed and disseminated
- ☐ Analyze evaluation data to inform second training
- ☐ Select trainers for collaborative research workshop
- ☐ Develop curriculum for second collaborative research training based on input from grantees
- ☐ Second collaborative research day-long training conducted and evaluated

☐ Grantees submit mid-way progress report
☐ Core staff review and provide feedback on progress reports
☐ Communication with and mentoring support to grantees throughout

2nd Half of Grant Program Period (August - December, 2019)

☐ Grantees continue implementation of collaborative research project
☐ Communication with and mentoring support to grantees throughout
☐ Analyze evaluation data to inform stories of impact and possible publication

Grant Close-Out (January, 2020)

☐ Program period ends (December 31, 2020)
☐ Grantees submit final, year-end report
☐ Final evaluation data collected and analyzed
☐ Develop stories of impact from the grantees

Following Grant Close-Out (February, 2020)

☐ Third Grantees Meeting: Presentations and Feedback
☐ Core staff review Final Reports
☐ Overall program final report
☐ Develop article/paper for general and/or peer-review audience
