# Supplementary material for: Building the foundation for equitable and inclusive research: Seed grant programs to facilitate development of diverse CBPR community–academic research partnerships
Source: J Clin Transl Sci. 2022 Nov 24;7(1):e2. doi: 10.1017/cts.2022.495 (PMC9879886; doi:10.1017/cts.2022.495)
Supplement: Supplementary file 1 [file S2059866122004952sup001.zip › S2059866122004952sup004.pdf]

Small Planning Grant Program (SPG)
Grants Awarded 2011 - 2022
Project Title, Partners, Affiliation, Amount

Round 1 - 2011

Youth Experiences of Violence, Discrimination and Harassment **$3,000**
- Laura Hughes Ruth Ellis Center
- Louis F. Graham UM School of Public Health

Stepping Back to Move Forward: Enhancing the Process and Capacity of the Aya Collaborative **$4,000**
- Sharon Lapides Community and Home Supports, Inc.
- Barbara L. Brush UM School of Nursing

Round 2 - 2012

Enhancing the Community Health Information Infrastructure in Northwest Detroit: Partnership Development Project **$4,000**
- Jennifer Hurt National Kidney Foundation of Michigan
- Tiffany Veinot UM School of Information

Detroit Climate Action Collaborative Climate Change Action Plan **$2,500**
- Joel Heeres Detroiters Working for Environmental Justice
- Kimberly Hill-Knott Detroiters Working for Environmental Justice
- Larissa Larsen UM College of Architecture and Urban Planning

Nd'nibwaakaami - We Are Wise: Sacred Bundle! Pilot Grant **$4,000**
- Ashley Tuomi American Indian Health and Family Services
- Sandra Momper UM School of Social Worker

Text 911: Using text messaging to evaluate perceptions of health care services for acute, unscheduled care **$10,000**
- Zachary Rowe Friends of Parkside
- Tammy Chang UM Medical School
- Adam Sharp UM Medical School

Round 3 - 2013

The Bell Tenants Research Partnership "The Partnership" **$4,000**
- Debbie Nicolellis Neighborhood Services Organization

- Tia Cobb — Neighborhood Services Organization
- Rebecca Cheezum — Oakland University School of Health Sciences

Detroit Urban Native Health Collaborative **$4,000**

- Ashley Tuomi — American Indian Health and Family Services
- Sandra Momper — UM School of Social Work

Health Team Members' Perceptions of the Role of Community Health **$10,000**

- Megha Shah — RWJF Clinical Scholar
- Gloria Palmisano — CHASS

Round 4 - 2014

Detroit Homeless Health Collaborative **$2,000**

- Ron Beford — Interfaith Health & Hope Coalition
- Paul Kilgore — Wayne State College of Pharmacy and Health Sciences

Preventing Substance Abuse and Mental Health Problems among Detroit Hispanic Youth **$4,000**

- Angela G. Reyes — Detroit Hispanic Development Corporation
- David Cordova — UM School of Social Work

Delray Impact Project - Translational Research to Inform Policy & Planning Decisions **$4,000**

- Simone Sagovac — Southwest Detroit Community Benefits
- Stuart Batterman — UM School of Public Health

Engaging Small Business to Improve Health and Health Care in Detroit **$3,000**

- Shannon Saksewski — Detroit Regional Chamber
- Jeffrey T. Kullgren — VA Ann Arbor Healthcare System

Round 5 - Winter 2015

Forming a collaborative partnership to increase effectiveness and reach of chronic disease prevention and management services among Detroit's elderly **$4,000**

- Letty Azar — Detroit Area Agency on Aging
- Mary Janevic — UM School of Public Health

Community-Based Recovery and Reentry Research in Detroit **$2,750**

- Dwight Vaughter — Self-Help Addiction Rehabilitation, Inc.
- Juliette Roddy — UM-Dearborn Dept. of Health and Human Services

Round 6 - Summer 2015

Collaborative Approach to Community-based Research on Breastfeeding **$4,000**

- Kiddada Green — Black Mothers' Breastfeeding Association
- Angela Johnson — UM Medical School

Identifying Healthcare Priorities in Detroit Homeless Population **$4,000**

- Ron Bedford — Interfaith Health & Hope Coalition
- Paul Kilgore — Wayne State College of Pharmacy and Health Sciences

Health care needs of people with intellectual/developmental disabilities living in Detroit **$3,760**

- Carmen McIntyre — Detroit Wayne Mental Health Authority
- Steven Erickson — UM College of Pharmacy

Round 7 - Winter 2016

Heat waves, housing and health: increasing climate resiliency in Detroit **$5,000**

- Zachary Rowe — Friends of Parkside
- Marie O'Neill — UM School of Public Health

Development of a Hypertension Focused Community Advisory Board **$4,975**

- Lisa Mason — Greater Detroit Area Health Council
- Phillip Levy — Wayne State University School of Medicine

Round 8 - Summer 2016

Community-Academic Partnership to Engage Family Supporters in Ongoing Chronic Disease Management for Latino and African-American Adults in Detroit **$4,997**

- Ricardo Guzman — CHASS
- Ann-Marie Rosland — UM Medical School

Round 9 - February 2018 - January 2019

Exploring Research Partnership Opportunities: Youth Development
$5,000
Programming Impact on Neighborhood Social Conditions

- Christine Bell — Urban Neighborhood Initiatives
- Robin Jacob — UM Youth Policy Lab

Partnership for Transgender Health and Resilience in Detroit **$5,000**

- Bre Ann Campbell — Trans Sistas of Color Project
- Gary Harper — UM School of Public Health

Increasing Purpose in Life among At-Risk Latino Adolescents **$5,000**

- Angela G. Reyes Detroit Hispanic Development Corporation
- Ruben Martinez Julian Samora Research Institute, Michigan State University

Partnership Formation in Community-Engaged Work: The Detroit Food Academy **$5,000**

- Jen Rusciano Detroit Food Academy
- Emily Nicklett UM School of Social Work

Round 10 - February 2019 - January 2020

Addressing a Critical Gap: Perceptions of Young African American Men
$5,000
of their Access to Sexual Health Services in a Community-Based Setting

- Wayne Bradley Detroit Community Health Connection
- Jade Burns UM School of Nursing

Promoting Infant Health and Wellbeing by Engaging Fathers in Home Visitation **$5,000**

- Nickole Fox American Indian Health & Family Services of Southeastern Michigan, Inc
- Shawna Lee UM School of Social Work

Development of the Healthy Chandler Park Community Advisory Board **$5,000**

- Yvette Bass Chandler Park Conservancy
- Phillip Levy Wayne State University School of Medicine

Round 11 - February 2020 - January 2021

Expanding a Collaborative Partnership to Support Trans-Masculine People of Color in Detroit **$5,000**

- Rimonte Parker Nuii Waav Brotherhood
- Elliot Popoff UM School of Public Health
- Gary Harper UM School of Public Health

Alley Activation as a Green Health Intervention **$5,000**

- Korey Batey Detroit Ain't Violent It's Safe (DAVIS)
- Paul Draus UM Dearborn

Expanding Youth Voice in Quality Assessment in Detroit: A
$5,000
Collaborative Project to Pilot and Disseminate a Youth-Led Evaluation

Tool (end May 1, 2022)

- Alicia McCormick — Urban Neighborhood Initiatives
- Sara Plachta Elliott — Youth Development Resource Coalition
- Katie Richards-Schuster — UM School of Social Work

The LGBTQ Senior Housing Project (End February 2022) **$5,000**

- Angie Perone — SAGE Metro Detroit
- Beth Glover Reed — UM School of Social Work

Building a Diverse Aging Network for Research, Service, and Knowledge Development in Detroit (End April 2022) **$5,000**

- Vincent Tilford — Hannan Center
- James Blackburn — UM School of Social Work

Round 12 - 2022

Tuxedo Project Community Map **$5,000**

- Rose Gorman — The Tuxedo Project
- Maureen Okasinski — UM School of Social Work

Operationalizing Videovoice to Capture and Communicate lived Experiences of Safety and Surveillance among Eastside Detroiters **$5,000**

- Zachary Rowe — Friends of Parkside
- Tawanna Dillahunt — UM School of Information

Welcome to the Motor City: Exploring Refugee Resettlement among Afghan Refugees in Detroit and Beyond **$5,000**

- Shadin Adityeh — Jewish Family Services of Washtenaw County and Detroit
- Ashley Cureton — UM School of Social Work and School of Education

Enacting Action Goals informed by HOMES Survey: Services and Options for LGBTQ+ Older Adults in Metro Detroit **$5,000**

- Angela Gabridge — Sage Metro Detroit
- Beth Glover Reed — UM School of Social Work

Investigating stream water contamination and prioritizing green Stormwater infrastructure in the River Rouge Watershed from a socioecological perspective **$5,000**

- Matthew Bertrand — Friends of the Rouge
- Runzi Wang — UM School for Environment and Sustainability
