# Supplementary material for: Building the foundation for equitable and inclusive research: Seed grant programs to facilitate development of diverse CBPR community–academic research partnerships
Source: J Clin Transl Sci. 2022 Nov 24;7(1):e2. doi: 10.1017/cts.2022.495 (PMC9879886; doi:10.1017/cts.2022.495)
Supplement: Supplementary file 1 [file S2059866122004952sup001.zip › S2059866122004952sup005.pdf]

2021 COMMUNITY-ACADEMIC RESEARCH PARTNERSHIPS
REQUEST FOR PROPOSALS
RESEARCH ON STRATEGIES TO PREVENT AND ALLEVIATE POVERTY IN MICHIGAN

DEADLINE FOR RECEIPT OF PROPOSAL: Wednesday, November 18, 2020

Purpose
Poverty Solutions at the University of Michigan and the Detroit Community-Academic Urban Research Center (Detroit URC) announce a grant funding opportunity for collaborations between academic researchers at the University of Michigan, Ann Arbor, Dearborn and Flint campuses, and community-based partners throughout the State of Michigan. The grants will support research projects focused on evaluating and strengthening interventions, programs, and policies that seek to prevent and alleviate poverty in the State of Michigan. A total of $80,000 is available for grants up to $20,000-$25,000 to undertake research that is collaboratively developed by community and academic partners. A faculty member from any U-M campus must serve as a Principal Investigator. Partnerships with faculty across multiple campuses are encouraged.

Background
Poverty Solutions is an inter-disciplinary initiative at the University of Michigan (U-M) that seeks to find new ways to prevent and alleviate poverty in Michigan, the nation and the world. The Detroit URC is a community-based participatory research (CBPR) partnership aimed at understanding and addressing the social and physical environmental factors that contribute to health inequities. Detroit URC equitably includes academic researchers at the University of Michigan and community-based organizations and health services agencies in Detroit. Poverty Solutions and the Detroit URC are working in partnership to support collaborative research in the State of Michigan that:

- enhances knowledge of the efficacy of interventions, programs and policies for preventing and alleviating poverty;
- equitably involves community and academic partners in all aspects of the research process;
- recognizes the strengths that each brings to the partnership;
- promotes co-learning and capacity building of all partners; and
- benefits community and academic partners.

Projects can support activities designed to prevent and alleviate poverty such as:

- an evaluation of an existing intervention, program or policy;

- an analysis of new or existing data to better understand the effectiveness of, or to strengthen, strategies (e.g., interventions, programs and policies);

- the development, implementation and preliminary evaluation of a new pilot program or an adaptation of an existing program; and

- an assessment of community needs, strengths, and resources that leads to the identification of new strategies.

Terms of Funding and Eligibility Criteria

The following terms apply:

1. Applicant teams are those addressing poverty in Michigan communities and include at least one U-M academic researcher and one community partner. Academic partners can be research professors, research scientists, or tenure track faculty. Community partners can represent nonprofit community-based organizations, government agencies, federally qualified health centers, and health and human services agencies.

2. Grants will begin January 1, 2021. Grants will end December 31, 2021. A midway progress report is due no later than July 31, 2021. A final report is due no later than January 31, 2022.

3. All team members are required to attend a virtual one-day grantees meeting on January 29, 2021. Facilitated by the Detroit URC, the meeting will address the development, implementation, and evaluation of collaborative research partnerships, and will include team presentations and working sessions.

4. During the second quarter of the grant period, all team members will attend a second one-day grantees meeting in July of 2021. Facilitated by a community-academic team, the meeting will address data interpretation and dissemination within the context of collaborative research partnerships, and will include team presentations, feedback, and working sessions.

5. In February of 2022, teams will attend a final, half-day session to report results, share accomplishments and lessons learned, and obtain feedback from peer teams of community academic researchers.

6. Project teams are strongly encouraged to publish and disseminate results in the peer-reviewed literature and more broadly. Recognizing that different partnerships will be at different stages of development; teams are expected to share either a published product or a plan and timeline for publishing products at the February 2022 session. Published products may include: 1) a paper submitted to the Poverty Solutions Working Paper Series, 2) a presentation of results at a conference or academic forum, 3) a grant proposal for continued funding related to the original

project, or 4) a peer-reviewed publication. Research teams are also strongly encouraged to disseminate results through policy briefs, community meetings, town hall meetings, fact sheets and local media outlets.

Selection Criteria

Applications are evaluated by academic and community partners affiliated with the Detroit URC and Poverty Solutions who have expertise in collaborative research and poverty prevention and alleviation. The selection criteria used to review the proposals include:

- the degree to which the study builds knowledge that informs the understanding of interventions, programs, or policies that seek to prevent or alleviate poverty;

- the quality of the research design, including appropriate research methodologies; · the feasibility of the proposed study to be completed during the funding period; · relevance of the project to the local community; and

- extent and feasibility of community and academic partner involvement.

Additional Criteria:

While not a requirement, preference is given to applications that meet the above criteria and one or two additional criteria:

- incorporation of U-M students into the project; and/or
- inclusion of multi-campus research teams.

Application Instructions

Applicants should submit their proposal via email to detroiturc@umich.edu. **Proposals must be received by 11:59 PM Eastern Standard Time on Wednesday, November 18, 2020**. Submit proposals as a single PDF file that includes the following elements in the order listed below.

1. A **cover sheet** with:
    a. The title of the proposed research project;
    b. Researcher and community partner names and organizational affiliations to include mailing addresses, email addresses, phone, and fax numbers;
    c. Name of the university and lead corresponding principal investigator (PI), who will be treated as the primary point of contact; and
    d. Contact information for a grant manager or financial administrator from the PI's department.

2. A one-page, double-spaced **abstract** describing the project's specific aims, data and methods, and relevance of the project to informing strategies to prevent and alleviate poverty. Explicitly connect research to future intervention or program implications.

3. A **project narrative** of no more than seven (7) double-spaced pages (excluding figures and references). The narrative should include sections that:
    a. describe the specific aims of the study (1 page),
    b. briefly summarize the relevant literature (1 page),

c. present major hypotheses or research questions (½ page),
d. describe research/intervention design, proposed methodology and data sources (2 pages),
e. describe extent of community involvement in identifying the focus and approach (½ page),
f. explain the role of community partners throughout the research process (½ page),
g. indicate how the proposed project will build knowledge about the efficacy of interventions, programs and/or policies that seek to address poverty (1 page), and
h. describe how results will be disseminated in peer-review literature and more broadly (½ page).

4. Considering frequency, location and structure of collaborative work and partnership, include a one-page, double-spaced **description** of:
    a. the experience and expertise that both community and academic partners bring to the project,
    b. any prior history the partners have collaborating on research projects, and
    c. evidence of the feasibility to accomplish the proposed aims.

5. An **itemized budget** and a **budget narrative** that explains each line item, with a minimum of 25% of funds designated for community partner organization(s). The department of the primary university investigator will serve as the fiduciary. Please detail each funding item requested. Appropriate research expenses include:
    a. Personnel (e.g., community and academic investigators, staff, student research assistants)
    b. Consultants (e.g., stipends for community partner organization(s))
    c. Project-related travel
    d. Hosting (e.g., for community meetings/steering committee meetings)
    e. Supplies, copying, printing

6. A **project timeline** listing specific milestones for study completion. The timeline must be within the period from January 1, 2021 through December 31, 2021.

7. **Curriculum vitae** for all academic researchers. **Biographies or resumes** for all community partners.

8. A **letter of support/commitment from a U-M department**, specifically accepting administrative responsibility for managing the grant.

9. A **letter of support/commitment from each community partner organization**, which specifies the interest and commitment to engage in the collaborative research project.

10. **Human subjects review approval** (often a waiver for secondary data analysis) is required for all projects before funding is dispersed.

Timeline of Important Dates

- Deadline for Receipt of Proposals: November 18, 2020
- Notification of Award: December 16, 2020

- Project Start Date: January 1, 2021
- First Grantees Meeting: January 29, 2021
- Second Grantees Meeting: July 2021
- Mid-Way Progress Report Due: July 2021
- Project End Date: December 31, 2021
- Final Report Due: January 31, 2022
- Third Grantees Meeting: February 2022

Contact Information
Direct questions to:
Detroit URC Center Manager
detroiturc@umich.edu
