# Supplementary material for: Building the foundation for equitable and inclusive research: Seed grant programs to facilitate development of diverse CBPR community–academic research partnerships
Source: J Clin Transl Sci. 2022 Nov 24;7(1):e2. doi: 10.1017/cts.2022.495 (PMC9879886; doi:10.1017/cts.2022.495)
Supplement: Supplementary file 1 [file S2059866122004952sup001.zip › S2059866122004952sup006.pdf]

Research on Strategies to Prevent & Alleviate Poverty in Michigan

Community-Academic Research Partnerships 2021 (Round 5)

Pre-Assessment

Thank you for participating in the Research on Strategies to Prevent & Alleviate Poverty in Michigan *Community-Academic Research Partnerships Grants* program. Please answer the following questions to assist in evaluating the overall effectiveness of the grants program, and to provide feedback to help guide and improve the program. Please complete the survey by **Wed., February 24**. The survey should take about 5-10 minutes of your time. Your answers will be kept confidential, and responses will be reported as a group so that no individual can be identified. Your participation is voluntary and filling out this survey indicates your consent. Thank you for your time!

1. For the purpose of this partnership, please choose which role you identify primarily as:
   - o Community partner
   - o Academic partner

2. Please indicate your experience working in community-academic collaborative research partnerships:
   - o This is my first experience
   - o I have some experience
   - o I have a great deal of experience

   - o Optional comments _____________

3. For each of the following, please rate your current level of *competence* in using a collaborative community-academic research approach to:

   (Scale: 1=None, 2=Low, 3=Moderate, 4=High, 5=Very high)

   - Build relationships between our community and academic research partners
   - Develop a steering committee or partnership infrastructure mechanism
   - Design and conduct collaborative research and/or interventions
   - Analyze data collaboratively
   - Feedback and interpret research findings within the partnership
   - Apply research findings to inform interventions and policies
   - Disseminate research findings to academic audiences
   - Disseminate research findings to non-academic (e.g., community, policy) audiences
   - Evaluate partnership process
   - Enhance equity among the partners
   - Develop new relationships outside of the partnership
   - Explore mutual shared research interests for future research collaboration
   - Submit grant proposals for collaborative research
   - Other (please describe) ________________

4. Please rate how important it is to you that your team accomplish the following during the next year of your project:
   Scale: Not important, Somewhat important, Moderately important, Very important, Extremely important, Not Applicable
   - Build relationships between our community and academic research partners
   - Develop a steering committee or partnership infrastructure mechanism
   - Design and conduct collaborative research and/or interventions
   - Analyze data collaboratively
   - Feedback and interpret research findings within the partnership
   - Apply research findings to inform interventions and policies
   - Disseminate research findings to academic audiences
   - Disseminate research findings to non-academic (e.g., community, policy) audiences
   - Evaluate partnership process
   - Enhance equity among partners
   - Develop new relationships outside of the partnership
   - Explore mutual shared research interests for future research collaboration
   - Submit grant proposals for collaborative research
   - Other (please describe) _________________

5. How likely it is that your partnership will achieve the following components of collaborative community-academic research during the next year: *Scale:* not at all likely, low likelihood, moderately likely, highly likely, extremely likely, not applicable
   - Build relationships between our community and academic research partners
   - Develop a steering committee or partnership infrastructure mechanism
   - Design and conduct collaborative research and/or interventions
   - Analyze data collaboratively
   - Feedback and interpret research findings within the partnership
   - Apply research findings to inform interventions and policies
   - Disseminate research findings to academic audiences
   - Disseminate research findings to non-academic (e.g., community, policy) audiences
   - Evaluate partnership process
   - Enhance equity among the partners
   - Develop new relationships outside of the partnership
   - Explore mutual shared research interests for future research collaboration
   - Submit grant proposals for collaborative research
   - Other (please describe) _________________

6. What is your greatest hope or aspiration for your partnership over the next year?

7. If there are any comments or additional information you'd like to provide, please feel free to do so here.

Thank you for completing this questionnaire!
Your input is important for assessing and improving the
Community-Academic Research Partnership Grant Program.

Direct any questions to: Detroit URC Center Manager, detroiturc@umich.edu or visit detroiturc.org
