# Supplementary material for: Building the foundation for equitable and inclusive research: Seed grant programs to facilitate development of diverse CBPR community–academic research partnerships
Source: J Clin Transl Sci. 2022 Nov 24;7(1):e2. doi: 10.1017/cts.2022.495 (PMC9879886; doi:10.1017/cts.2022.495)
Supplement: Supplementary file 1 [file S2059866122004952sup001.zip › S2059866122004952sup007.pdf]

*Detroit URC & Poverty Solutions Community-Academic Grant Program*

*Research on Strategies to Prevent and Alleviate Poverty*

# Application Review Process

LAST UPDATED: 11-24-20

The following process will be utilized to review grant proposals submitted to the Detroit URC & Poverty Solutions Community-Academic Grant Program.

1. Detroit URC staff will screen all submitted applications for completeness using the *Community-Academic Grants Triage form*. Applicants will be contacted if their grant proposal is not complete. They will have 48 hours to resubmit.
2. All successfully submitted grant proposals will be distributed evenly among the entire Review Committee to evaluate (2 to 5 proposals per reviewer, depending on number of proposals received). Each member of the Review Committee will review their assigned applications using the *Community-Academic Grant Proposal Review Form* to assess if teams and proposed projects would be a good fit for the program. Each proposal will be reviewed by two primary reviewers (at least one community-based and one academic reviewer).
    a. On the score sheet, Review Committee members will score proposals on 13 criteria and provide an overall recommendation for each application using a five-point scale [“Very Weak” to “Very Strong”].
    b. Recusal Process: Review Committee members will not score grants for applicants if the Reviewer has co-authored papers or applied for grants with the applicant, or if the Reviewer and applicant have a strong personal connection. However, the Reviewer may participate in a group discussion on the applicant’s proposal. If reviewers receive a grant proposal for which they need to recuse themselves, they will notify via email Carol Gray at ccbgray@umich.edu. She will send the Reviewer an alternative application to review.
3. The Review Committee will meet to reach consensus on which applications will be chosen.
    a. Prior to meeting, Detroit URC staff will compile all comments and scores so that each review member can view compiled comments and scores. The Review Committee will also receive copies of all the proposals as background information and to refer to during group discussion.
    b. At the meeting, the Review Committee will initially decide if there are any teams that should clearly be chosen/or clearly not be considered (based on the overall recommendation of the two primary reviewers).
    c. The Review Committee will discuss all other applications according to reviewers’ preferences.
    d. The Review Committee will make final decisions by consensus, using the 70% agreement rule.
4. Detroit URC staff will notify applicants of award decisions, and cc the Poverty Solutions team.

## Timeline

| Date | Description |
|---|---|
| **November 18** | Application Deadline |
| **November 19** | Detroit URC staff screen applications for completeness |
| **November 24** | Detroit URC staff send applications to Review Committee members |
| **December 7** | Reviewers complete application review and send responses on Proposal Review Form to Carol |
| **December 8** | Detroit URC staff compile scores and comments into a common spreadsheet |
| **December 8, 9, 10, 11** | Reviewers and Detroit URC staff members meet to make final decisions |
| **December 14, 15, 16** | Detroit URC staff notify applicants |
| **Early January 2021** | Coordinated media announcements released by Poverty Solutions and Detroit URC |

For more information or to learn more about the work of the Detroit URC, the CAR-Network, and other community-academic capacity building opportunities visit: www.detroiturc.org.

For more information about Poverty Solutions, visit: https://poverty.umich.edu/
