# Supplementary material for: Building the foundation for equitable and inclusive research: Seed grant programs to facilitate development of diverse CBPR community–academic research partnerships
Source: J Clin Transl Sci. 2022 Nov 24;7(1):e2. doi: 10.1017/cts.2022.495 (PMC9879886; doi:10.1017/cts.2022.495)
Supplement: Supplementary file 1 [file S2059866122004952sup001.zip › S2059866122004952sup008.pdf]

October 11, 2021

Availability of Small Planning Grants to Foster New Collaborative Research in Detroit

Summary

The Detroit Community-Academic Urban Research Center (Detroit URC) announces its twelfth round of small planning grant funding. This program aims to foster the establishment of new community-academic research partnerships and new collaborative research efforts in Detroit through two types of grants: 1) Partnership Formation & Maintenance Grants, and 2) Project Development Grants. **Full proposals are due December 10, 2021**.

The **Detroit URC** and the **Michigan Institute for Clinical & Health Research (MICHR) Community Engagement (CE) Program** are helping to support this Small Planning Grant Program in Detroit. In addition, partnerships with **U-M School of Social Work** faculty will be supported by the U-M School of Social Work.

For partnerships not based in Detroit, funding is available through MICHR. For more details on current funding opportunities, visit MICHR's website at michr.umich.edu.

What is the Detroit Community-Academic Urban Research Center?

The Detroit URC is a community-based participatory research (CBPR) partnership, which involves community-based organizations, practice-based and government agencies, and health service institutions in Detroit and academic researchers at the University of Michigan. Since 1995, the Detroit URC and its affiliated partnerships have been working to foster and support CBPR partnerships in order to gain a better understanding of the social and physical environmental determinants of health and to reduce and, ultimately, eliminate health inequities in Detroit. Our long-standing partnership has allowed us to establish close ties and excellent working relationships among our Detroit-based partners. For a list of partner organizations and affiliated partnerships and for more information about the Detroit URC, including additional resources on CBPR, please visit the Detroit URC website at www.detroiturc.org.

What is Community-Based Participatory Research (CBPR)?*

*CBPR is one approach for collaborative research

CBPR Key Components:

- Involves equitably all community and academic partners in all aspects of the research process, recognizes the unique strengths that each brings, and promotes co-learning and capacity building of all partners;
- Begins with a research topic of importance to the community;
- Combines knowledge with programs and policies to improve health and well-being and reduce health inequities; and
- Benefits community and academic partners through:

- Improved understanding of the communities involved,
- Enhanced relevance and use of the research,
- Enhanced quality and validity of data, and
- Improved lives of the people involved.

What is the Small Planning Grant Program Purpose and Scope?

The Small Planning Grant Program seeks to facilitate new, equitable collaborative relationships between academic and community entities and enhance their capacity to conduct and disseminate research focused on the social and physical environment and health equity, and translate research findings into action, with the aim of improving the health and quality of life of Detroit residents.

What Types of Projects Will This Program Fund?

The Detroit URC Small Planning Grant Program will award two types of grants as described below.

1) Partnership Formation & Maintenance Grants

These grants are intended to support newly developing or potential partnerships in, for example, relationship building, exploration of shared areas of interest, creation of partnership structure, and identification of potential research collaborations. Examples of activities that would be funded by Partnership Formation & Maintenance Grants include:

- **building relationships between partners** (e.g., facilitating formal and informal meetings for partners to learn more about each other, to explore the involvement of other potential partners, and to discuss how the partners will adopt and use equitable collaborative principles and operating norms);
- **exploring shared research interests and identifying capacity building needs** (e.g., identifying partnership research priorities, determining nature of collaborative research approach that will be used, delineating capacity-building needs, participating in trainings and/or ongoing mentoring opportunities); and
- **developing a Steering Committee and/or other appropriate partnership infrastructure mechanisms** (e.g., defining roles and responsibilities; developing communication and decision-making mechanisms, policies and procedures; developing a set of collaborative participatory research principles to guide decisions regarding various aspects of the research process).

2) Project Development Grants

These grants are intended to support established partnerships in, for example, the development of specific new collaborative research efforts, analysis of existing data, enhancing capacity for conducting collaborative research, the dissemination and translation of research findings, and the evaluation of the partnership process. Examples of activities that would be funded by Project Development Grants include:

- **conducting community assessment(s) to inform new collaborative research efforts** (e.g., focus groups to gather data to inform the research question and/or methods);
- **analyzing existing data to help inform future collaborative research** (e.g., collaborative analysis, review and interpretation of previously collected data);
- **disseminating and translating research findings** (e.g., development and implementation of appropriate dissemination tools, such as, fact sheets and policy briefs, to community and academic audiences and to policy-makers);
- **planning future research collaboration and proposal submissions** (e.g., identifying and responding to potential funding opportunities); and
- **evaluating the partnership process** (e.g., collecting data to assess how and to what extent the partnership is achieving its goals, and the challenges and facilitating factors associated with developing, maintaining and sustaining the partnership, and identifying future directions for the partnership based on the assessment conducted).

What Is the Funding Level and How Long Is the Project Period?

Small planning grants may be funded up to a maximum amount of $5,000 with a grant period duration of up to one year. Between 4 to 5 awards may be made this round. The final number and amount of awarded grants will be based on the quantity and quality of the submitted proposals. The proposal's budget and justification should match the requested project amount and timeline. Applicants will be notified by email about funding decisions.

Who Can Apply for Small Planning Grants?

Proposals may be submitted by academic researchers and representatives from community organizations who are committed to engaging in collaborative research in Detroit as described above. Applications must be submitted on behalf of at least two partners: an academic partner affiliated with an academic institution, and a community partner representing a non-academic community-based organization, practiced-based organization, or government agency in Detroit.

How Will Proposals Be Evaluated?

Grant applications will be reviewed by a committee composed of faculty and staff from the Detroit URC, MICHR and SSW, and both community and academic partners with extensive experience in and understanding of community-based participatory research. The Detroit URC is seeking proposals with the potential for creating new or developing existing effective collaborative research efforts that will address the social and physical environmental factors that contribute to health inequities of relevance to Detroit communities.

Partnership Formation & Maintenance Grant selection criteria are:

- Evidence of the rationale for the need for this collaborative
- Evidence of responsiveness to the community partner's needs/interests/strengths
- Extent and feasibility of community and academic partner involvement
- Potential for future research collaboration
- Potential of funding for future collaborative research effort(s)
- Clear, detailed and reasonable budget
- Clear, detailed and realistic timeline
- Evidence that the proposal and budget have been developed and discussed by all partners with funding allocated equitably as appropriate

Project Development Grant selection criteria are:

- Relevance of proposed research effort to Detroit communities involved
- Extent and feasibility of community and academic partner involvement
- Extent to which basic collaborative research principles are applied through the current project and in past efforts
- Evidence of responsiveness to the community partner's needs/interests/strengths
- Potential positive impact on the goals of the project (e.g., new research priorities identified, dissemination materials developed, capacity building workshops conducted, partnership process improved)
- Potential of funding for future collaborative research effort(s)
- Clear, detailed and reasonable budget
- Clear, detailed and realistic timeline
- Evidence that the proposal and budget have been developed and discussed by all partners with funding allocated equitably as appropriate

Post-Award Requirements

All grantees will participate in an half-day orientation and working session on conducting collaborative research. Grantees will be required to submit a mid-point progress report and a final project and budget report, and participate in completing evaluation questionnaires. If not already members of the Detroit URC Community-Academic Research Network (CAR-Network), a virtual entity of local community organizations, health and human service agencies, and academic researchers, grantees will become members of the CAR-Network.

Post-Award Capacity-Building Opportunities

Grantees will have access to participate in capacity building opportunities offered by the Detroit URC such as training workshops and/or technical assistance and mentoring in collaborative research.

How to Apply

Please download the Small Planning Grant application form from the Detroit URC Small Planning Grant webpage. Detailed instructions regarding contact information and submitting your application are included on the form. All applications must include the following (see form for more specificity):

1. **Application Cover Page** (Contact and Organization Information) (1-page)
2. **Proposal Narrative** (project rationale, goals/objectives, timeline, proposed outcomes) (between 4-5 pages)
3. **Budget and Justification** (last page of application packet) (up to 2 pages)
4. **Letter(s) of Commitment:** One from each of the participating community organization(s) involved and one from each of the participating academic researcher(s) involved, which address the interest and commitment of the partners to engage in the proposed collaborative research effort. (up to 2 attachments)
5. (Optional) Up to 5 additional items that illustrate the intent to establish a participatory collaborative research partnership or the degree to which such a partnership is already established may be included as appendices with the application submission.

Format Specifications: Please type applications in Arial Font and Font Size 11. Text should be single spaced on standard size (8.5” x 11”) paper.

How to Find More Information

For more information please contact: Katie Corbit Detroit URC Center Manager, at khcorbit@umich.edu.

Planning Grant Program Round 12 Timeline:

Planning Grant Announcement	October 11, 2021
**Proposal Submission Deadline**	**December 10, 2021**
Anticipated Notice of Awards	January 17, 2022
Anticipated Award Start	February 1, 2022

The Detroit URC Small Planning Grant Program is supported by the Detroit URC and the Michigan Institute for Clinical & Health Research (MICHR), and partnerships with U-M School of Social Work faculty will be supported by the U-M School of Social Work.

For more information or to learn more about the work of the Detroit URC, the CAR-Network, and other community-academic capacity building opportunities visit: www.detroiturc.org.
