# Supplementary material for: Building the foundation for equitable and inclusive research: Seed grant programs to facilitate development of diverse CBPR community–academic research partnerships
Source: J Clin Transl Sci. 2022 Nov 24;7(1):e2. doi: 10.1017/cts.2022.495 (PMC9879886; doi:10.1017/cts.2022.495)
Supplement: Supplementary file 1 [file S2059866122004952sup001.zip › S2059866122004952sup009.pdf]

# *Detroit URC & University of Michigan Poverty Solutions Community-Academic Grant Program: Project Questionnaire*

The purpose of this brief survey of participants from [Round #] of our Poverty Solutions Community-Academic Grant Program funding is to understand to what extent seed funding impacts grantee partnerships beyond the conclusion of the funding period. Your participation is voluntary and completing the survey indicates your consent. Your answers will be kept confidential, and the results will be reported in summary form in such a way that no individual participant can be identified. The questionnaire takes approximately 10 minutes and can be saved and continued if you're unable to complete it in one sitting. We greatly appreciate your responses to this questionnaire.

**Q1. Which of the following describe the status of the partnership that was supported by the Community-Academic Grant Program? Check all that apply:**

a. Our partnership is continuing to work on the same project.
b. The partnership has ended its work on our initial project.
c. Our partnership is working together on a different project.
d. Our partnership is no longer working together.
e. The initial partnership continues although some of the partners have changed.
f. In part due to this program, the partner/s involved have gone on to establish other CBPR partnership/s.

**Q2. Which of the following are accomplishments of your partnership that was supported by the Community-Academic Grant Program: Check all that apply.**

a. Established or strengthened formal partnership using a CBPR approach
b. Collected and interpreted data using a CBPR approach
c. Completed a research project using a CBPR approach
d. Established priorities for CBPR projects
e. Developed an intervention/program
f. Conducted an intervention/program
g. Worked on a policy change effort based on the research findings.
h. Evaluated the partnership
i. Conducted a training/workshop on CBPR
j. Disseminated the work of the partnership to community audiences
    a. Presentations
    b. Website/s
    c. News articles
    d. Reports
    e. Social media
    f. Other _____________
k. Disseminated the work of the partnership to academic audiences
    a. Oral presentation at a professional meeting or conference
    b. Poster presentation at a professional meeting or conference
    c. Prepared an article for publication
        i. If checked, Status of the article:

1. Submitted
2. Under review
3. Revised and resubmitted
4. Accepted, published
    a. If published, please include the title of all articles published and the journal name(s)________________________________

l. Submitted grant proposal/s:
    a. If checked, How many grant proposals did you submit?_________________________

m. Received funding (internal or external) to extend the work of the partnership.
    a. If checked, please tell us the following:
        i. Grant/Project Title _____________________
        ii. Funder Name ________________________
        iii. Amount Funded _______________________
        iv. Funding Period ________________________________

**Q3. Please share any additional comments regarding participating in the Community-Academic Grant Program.**

| |
|---|
| |

**Q4. Please select which best describes who completed this questionnaire:**

**a.** Community partner
**b.** Academic partner

**Q5. May we reach out to you by email if we have any questions?**

**a.** Yes
    **a.** If checked, please provide an email address. ______________________________
**b.** No

**Thank you for completing this questionnaire, and for your ongoing contributions! If you have any questions, please contact us at DetroitURC@umich.edu.**
