# Supplementary material for: Building the foundation for equitable and inclusive research: Seed grant programs to facilitate development of diverse CBPR community–academic research partnerships
Source: J Clin Transl Sci. 2022 Nov 24;7(1):e2. doi: 10.1017/cts.2022.495 (PMC9879886; doi:10.1017/cts.2022.495)
Supplement: Supplementary file 1 [file S2059866122004952sup001.zip › S2059866122004952sup010.pdf]

Research on Strategies to Prevent & Alleviate Poverty in Michigan
12 Month Progress Report Form
Due: September 13, 2021

Please complete and submit this report via email to xxx. Please limit your responses so that the total length of your narrative (not including your budget report or attached materials) is no longer than 2-3 pages in length. Thank you.

1. **Title:** Please list the title and the community and academic primary investigators *(name, title, and organization).*

2. **Collaborative Approach:** Please describe how you used a collaborative approach to achieve your stated goals and objectives, including roles of community and academic partners and what each has brought to the project.

3. **Results:** Please describe the outcomes/results of your project/partnership based on your stated goals and objectives. If relevant, describe any impacts (broad or longer term effects, such as health, environment, systems, or policy change). If the project is not complete, please describe plans for completing any objectives that are still in progress.

4. **Challenges:** Describe any challenges you may have encountered in your efforts and how you have addressed them or plan to address them.

5. **Lessons Learned:** Please discuss lessons learned from the project/partnership, and how they will inform your future work.

6. **Publication/Dissemination:** What actions have been taken or are planned to disseminate your partnership work in the peer-reviewed literature, for community audiences, or more broadly? Published products may include papers, presentations, forums, policy briefs, fact sheets, and media coverage. Please provide links or attach products.

7. **Sustainability:** Do you intend to sustain this project/partnership after the grant period has ended? If so, what steps have been taken or what actions will you take to facilitate sustainability (e.g., pursuit of future funding opportunities)?

8. **Budget Report**: Please indicate the final balance of your budget. Include receipts for items purchased. If the entire budget was not spent, please describe why.

Please attach any relevant documentation or project materials (e.g. photos, attendance sheets, evaluation forms, etc.)

Please direct questions to Detroit URC Center Manager:
detroiturc@umich.edu
