# Supplementary material for: Building the foundation for equitable and inclusive research: Seed grant programs to facilitate development of diverse CBPR community–academic research partnerships
Source: J Clin Transl Sci. 2022 Nov 24;7(1):e2. doi: 10.1017/cts.2022.495 (PMC9879886; doi:10.1017/cts.2022.495)
Supplement: Supplementary file 1 [file S2059866122004952sup001.zip › S2059866122004952sup011.pdf]

Community-Academic Research Partnership Grant Program:
Research Strategies to Prevent and Alleviate Poverty in Michigan

Mid-Way Progress Report Guidelines

Please answer the following questions to report on the progress of your research project to date. Use the below outline to organize the sections of your report. The total length of your narrative, not including the budget report, should be no more than 2-3 pages. Please send your team's report via email to detroiturc@umich.edu by **September 13, 2021**. Thank you!

1. **Title Page:** Be sure to include the project title, academic and community primary investigators, and date your report.

2. **Goals and Objectives:** Please provide an update on the status of achieving the goals and objectives of your project, including activities completed and/or in progress. If you've made any changes to your goals and objectives, please describe.

3. **Collaborative Approach:** Please describe how you are using a collaborative approach to achieve your stated goals and objectives, including roles of community and academic partners and what each has brought to the project.

4. **Challenges:** Describe any challenges you may have encountered in your efforts and how you have addressed them or plan to address them.

5. **Remaining Grant Period:** Please describe your plans for the next 6 months of the grant period.

6. **Dissemination**: We realize you are only six months into your project, however, if you have developed any plans for disseminating your work to community and academic audiences, or more broadly, please briefly describe them here.

7. **Sustainability**: Likewise, if your partnership has taken any steps toward sustaining itself and/or pursuing future funding opportunities, please describe.

8. **Assistance**: Please describe any areas that you would like to discuss with our team. As a reminder, the Detroit URC and Poverty Solutions are available to provide technical assistance such as: identifying additional grant funding, publicizing the project to a broad audience, and/or meeting to discuss ways in which we can be of support to your effort. Please do not hesitate to let us know if there are ways we can assist.

9. **Budget Report**: Please attach a budget report to your narrative that shows the balance of each line item in the budget you submitted with your proposal.

Please direct questions to Detroit URC Center Manager: detroiturc@umich.edu
