# Supplementary material for: Building the foundation for equitable and inclusive research: Seed grant programs to facilitate development of diverse CBPR community–academic research partnerships
Source: J Clin Transl Sci. 2022 Nov 24;7(1):e2. doi: 10.1017/cts.2022.495 (PMC9879886; doi:10.1017/cts.2022.495)
Supplement: Supplementary file 1 [file S2059866122004952sup001.zip › S2059866122004952sup012.pdf]

DETROIT URC
DETROIT COMMUNITY - ACADEMIC URBAN RESEARCH CENTER

POVERTY SOLUTIONS
UNIVERSITY OF MICHIGAN

Research on Strategies to Prevent & Alleviate Poverty in Michigan

Community-Academic Research Partnerships (Round 4) - Grantees Meeting #1

Thursday, January 30, 2020

Grantees Meeting Assessment Questionnaire

Thank you for participating in the Community-Academic Research Partnerships Grantees Meeting. Please answer the following questions to assist in evaluating the overall effectiveness of the grants program, and to provide feedback to help guide and improve the program. Your responses are confidential, and will be reported as a group so that no individual participant can be identified. We value your comments and look forward to continuing to working together!

1. For the purpose of this partnership, in which role do you primarily identify:
   - ☐ Community partner
   - ☐ Academic partner

2. Please indicate your level of agreement with the following statements about the Grantees Meeting by putting an X in the appropriate column:

	Strongly Disagree	Disagree	Neither Agree nor Disagree	Agree	Strongly Agree
Overall the meeting content and structure was well-organized.					
The presentation on CBPR and partnership development was useful.					
The presentation on partnership evaluation was useful.					
Meeting facilitators demonstrated expertise in the subject matter.					
Learning resources (binder, handouts, resource list) will be useful to our partnership in the future.					
Interactive exercises and questions were used effectively.					
Opportunities for our team to work together on specific tasks were valuable.					
Learning about and networking with other partnerships was beneficial to our work.					
	Not at all Useful	Somewhat Useful	Moderately Useful	Very Useful	Extremely Useful
Developing Operating Norms					
Partnership development					
Partnership Evaluation					
Partnership Next Steps					
Further development of working relationships among your team					

3. Please indicate how useful the Team Working Sessions were for your partnership in each of the following areas:

Comments:

(over)

4. What were the **most** valuable/beneficial aspects of the Grantees Meeting?

5. What were the **least** valuable/beneficial aspects of the Grantees Meeting?

6. What **recommendations** do you have on how we could improve the Grantees Meeting?

7. What, if anything, would you like to learn more about in order to most effectively develop your community-academic partnership and carry out your collaborative research project? Include any ways that the Detroit URC team may be of assistance as you move forward.

8. What would you like the next Grantees Meeting to focus on (to be held in May/June)?

9. If there is any additional feedback you'd like to provide, please feel free to do so here.

Thank you for completing this questionnaire! Your feedback is important for assessing and improving the Community-Academic Research Partnership Grant Program.

Please direct questions to Detroit URC Center Manager: detroiturc@umich.edu
