# Supplementary material for: Building the foundation for equitable and inclusive research: Seed grant programs to facilitate development of diverse CBPR community–academic research partnerships
Source: J Clin Transl Sci. 2022 Nov 24;7(1):e2. doi: 10.1017/cts.2022.495 (PMC9879886; doi:10.1017/cts.2022.495)
Supplement: Supplementary file 1 [file S2059866122004952sup001.zip › S2059866122004952sup013.pdf]

DETROIT URC
COMMUNITY - ACADEMIC
URBAN RESEARCH CENTER

Detroit URC Small Planning Grants
6 Month Progress Report Form

Please complete and submit this report via email to xxx. Please limit your responses so that the total length of your narrative (not including your budget report) is no longer than 2 pages in length. Thank you.

1. Please provide an update on the status of achieving the goals and objectives of your project development grant, including activities completed and/or in progress and any challenges you may have encountered in your efforts.

2. Please describe how you are using a collaborative/participatory approach to achieve your stated goals and objectives.

3. Please describe your plans for the next 6 months of your grant.

4. Please describe the steps your partnership has taken, if any, towards sustaining itself and/or pursuing future funding opportunities?

5. If you have developed any communication or dissemination materials or products (e.g., project description, one-pager, fact sheet, web page, article, presentation), please describe and/or attach.

6. If you have any specific technical assistance needs with which we might be able to help please let us know.

Please attach a budget report to your narrative which shows the balance of each of the line items in the budget you submitted with your proposal.

Thank you!
Please direct questions to Detroit URC Center Manager: detroiturc@umich.edu
