# Supplementary material for: Building the foundation for equitable and inclusive research: Seed grant programs to facilitate development of diverse CBPR community–academic research partnerships
Source: J Clin Transl Sci. 2022 Nov 24;7(1):e2. doi: 10.1017/cts.2022.495 (PMC9879886; doi:10.1017/cts.2022.495)
Supplement: Supplementary file 1 [file S2059866122004952sup001.zip › S2059866122004952sup014.pdf]

Community-Academic Research Partnership Grants Program (C-A)

Grants Awarded 2017 - 2021

Project Title, Partners, Affiliation, Amount

Round 1 - 2017

Barrier Busting in the HOPE Village Neighborhood Network **$25,000**

- Stephanie Moore UM Stephen M. Ross School of Business
- Noel Tichy UM Stephen M. Ross School of Business
- Julie Phenis Focus: HOPE
- Debbie Fisher Focus: HOPE

Helping a House Remain a Home **$24,994**

- Roshanak Mehdipanah University of Michigan School of Public Health
- Alexa Eisenberg University of Michigan School of Public Health
- Ted Phillips United Community Housing Coalition
- Michele Oberholtzer United Community Housing Coalition

Improving Health and Strengthening Communities **$24,203**

- Michele Heisler UM Institute of Healthcare Policy and Innovation (IHPI)
- David J. Law Joy-Southfield Community Development Corporation, Inc.(JSCDC)
- Abdul El-Sayed Detroit Health Department

Preserving Low-Income Housing in Detroit **$25,000**

- Margaret Dewar UM Taubman College of Architecture and Urban Planning
- Lan Deng UM Taubman College of Architecture and Urban Planning
- Sarida Scott Community Development Advocates of Detroit
- LaToya Morgan Community Development Advocates of Detroit
- Julie Schneider Detroit Department of Housing and Revitalization

Round 2 - 2018

Overcoming the chilling effect: Identifying strategies for improving immigrant families' acceptability and accessibility to health and social services that alleviate poverty **$30,000**

- Paul J. Fleming University of Michigan School of Public Health
- William D. Lopez University of Michigan School of Public Health
- Richard Bryce Community Health and Social Services Center (CHASS)

Partnership for Economic Independence **$30,000**

- Tawanna Dillahunt — UM School of Information
- Donna Givens — Eastside Community Network
- Angela Brown Wilson — Eastside Community Network

Breaking the Cycle: Refining the Trauma-Informed Clinical Ethnographic Narrative Intervention (CENITF) **$30,000**

- Laura E. Gultekin — University of Michigan School of Nursing
- Delphia Simmons — Coalition on Temporary Shelter

Assessing the impact of intergenerational asset building programming on the self-efficacy, academic achievement and college going culture of low-income Black and Latino girls **$30,000**

- Trina Shanks — University of Michigan School of Social Work
- Melody Moore — Alternatives for Girls

Round 3 - 2019

Engaging low-income and minority communities in prioritizing community health benefits **$25,000**

- Susan Dorr Goold — University of Michigan Medical School
- Zachary Rowe — Friends of Parkside

Providing opportunity, not punishment: implementing a pilot functional sentencing program in southeast Michigan **$24,208**

- Francine Banner — University of Michigan-Dearborn
- Jayesh Patel — Street Democracy

Michigan evictions: assessing data sources and exploring determinants **$25,000**

- Robert Goodspeed — UM Taubman College of Architecture and Urban Planning
- Margaret Dewar — UM Taubman College of Architecture and Urban Planning
- Elizabeth Benton — Michigan Advocacy Program

Identifying the recipe for success: can a new cooking class program in a community health center increase participation in existing center programs and build core skills to decrease food insecurity among low-income patients? **$25,000**

- Julia A. Wolfson — University of Michigan School of Public Health
- Caroline Richardson — University of Michigan Medical School
- Richard Bryce, D.O. — Community Health and Social Services Center (CHASS)
- Denise Pike, AICP — Community Health and Social Services Center (CHASS)

Round 4 - 2020

The Afro-Futurist Greenhouse

- Ron Eglash — UM School of Information
- Audrey Bennett — UM Penny W. Stamps School of Art and Design
- Olayami Dabls

Poverty Alleviation through Entrepreneurship and Urban Microenterprise Development

- Marcus D. Harris — University of Michigan-Dearborn College of Business
- Crystal J. Scott — University of Michigan-Dearborn College of Business
- Jeffrey Robinson — Rutgers Business School
- April Boyle — Build Institute
- Lashawna Manigault — LifeLine Business Consulting Services, LLC

Special Delivery: A Community Academic Partnership to Improve the Health of Low-Income Young Mothers and their Children

- Tammy Chang — University of Michigan Department of Family Medicine
- Gayathri Akella — Washtenaw County Health Department WIC
- Marika Waselewski — University of Michigan Department of Family Medicine

Round 5 - 2021

The "Community Tech Workers": A Community-Driven Model to Support Economic Mobility by Bridging the Digital Divide

- Zachary Rowe — Friends of Parkside
- Tawanna Dillahunt — UM School of Information
- Julie Hui — UM School of Information

Detroit Housing Counseling: Discovery and Community Data

- Hector Hernandez — Southwest Economic Solutions of Detroit
- David Palmer — David Palmer, LLC
- Trina Shanks — UM School of Social Worker
